# Prime Editing for Precision Genetic Medicine: A Systematic Review of Technologies, Delivery, and Therapeutic Applications

**DOI:** 10.3390/genes17091138

**Published:** 2026-09-17

**Authors:** Douglas M. Ruden

**Affiliations:** Institute of Environmental Health Sciences, Charles S. Mott Center for Human Growth and Development, Department of Obstetrics and Gynecology, Wayne State University, Detroit, MI 48201, USA; douglasr@wayne.edu; Tel.: +1-313-577-6688

**Keywords:** prime editing, CRISPR, precision genetic medicine, genome editing, pegRNA, gene therapy, delivery, therapeutic genome editing, genome writing

## Abstract

Background: Prime editing has rapidly evolved from a CRISPR-based “search-and-replace” approach for precise sequence modification into a diverse family of genome editing technologies. This systematic review maps the technological evolution of prime editing, with emphasis on editor architecture, guide RNA engineering, delivery, therapeutic applications, computational approaches, and emerging capabilities. Methods: PubMed and Web of Science were systematically searched for studies in which prime editing constituted a substantive experimental, technological, computational, delivery, or therapeutic component. After deduplication and screening, candidate studies underwent manual re-screening against prespecified eligibility criteria. Reviews, corrections, plant and bacterial studies, conventional CRISPR or base editing studies without a substantive prime editing component, and other non-relevant records were excluded. A total of 294 studies were included in the final systematic evidence synthesis. Because of substantial heterogeneity in editor architectures, targets, experimental models, outcomes, and reporting, the literature was synthesized using systematic mapping and qualitative thematic analysis rather than meta-analysis. Results: The evidence demonstrates rapid diversification from the original Cas9 nickase–reverse transcriptase–prime editing guide RNA architecture through improvements in pegRNA design, Cas and reverse transcriptase engineering, DNA repair modulation, delivery, computational design, and increasingly complex sequence modification. Therapeutic studies span disease modeling, correction of pathogenic variants, ex vivo applications, and direct in vivo editing; however, high editing efficiency does not necessarily translate into functional or therapeutic rescue. Large-sequence insertion and replacement strategies further extend the capabilities of prime editing, although these approaches remain less mature than small-sequence correction and face substantial challenges in efficiency, fidelity, cargo delivery, and genomic safety. Conclusions: Prime editing has developed into a versatile precision genome editing platform, but the evidence base remains heterogeneous and predominantly preclinical. Translation to genetic medicine will require improvements in reproducibility across targets and cell types, delivery to clinically relevant tissues, product purity, genomic safety, and demonstration of meaningful functional benefit. Emerging large-sequence editing approaches broaden the potential scope of prime editing but should be distinguished from technologies with established experimental and therapeutic evidence.

## 1. Introduction

The development of CRISPR-based genome editing has transformed experimental genetics and created new opportunities for correcting disease-causing genetic variants. Conventional CRISPR–Cas9 nucleases generate targeted DNA double-strand breaks (DSBs), which are subsequently resolved by endogenous DNA repair pathways. Although highly effective for gene disruption, DSB-dependent editing can generate heterogeneous insertion and deletion products and can be difficult to use for precise sequence replacement. Homology-directed repair (HDR) can introduce defined sequence changes but generally requires an exogenous donor DNA template and is strongly influenced by cellular DNA repair activity and cell cycle state. Base editing represented an important advance by enabling selected nucleotide substitutions without DSBs; however, conventional base editors are restricted in the classes of nucleotide changes they can directly install and may generate bystander edits. These limitations created a need for a more versatile system capable of precisely rewriting genomic sequence without requiring DSBs or conventional donor DNA templates.

**Figure 1:**
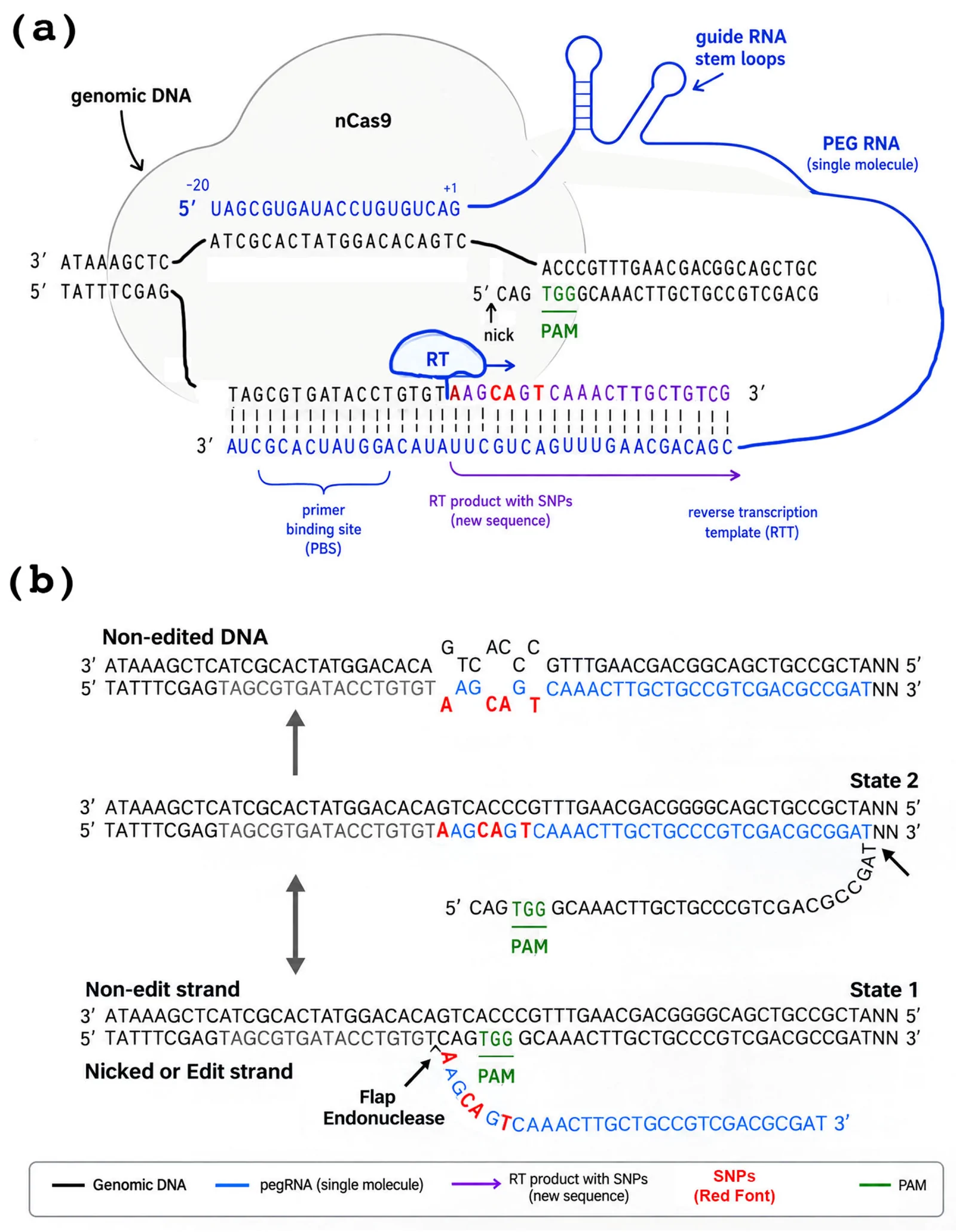
Molecular mechanism of prime editing. (**a**) Prime editing is mediated by a Cas9 H840A nickase (nCas9), reverse transcriptase (RT), and a single continuous prime editing guide RNA (pegRNA). The pegRNA contains the CRISPR targeting sequence and guide RNA stem loops together with a 3′ extension comprising the primer binding site (PBS) and reverse transcription template (RTT). Following target recognition, nCas9 nicks the edit strand adjacent to the protospacer-adjacent motif (PAM), generating a genomic 3′-DNA end that hybridizes to the PBS and serves as the primer for reverse transcription. RT then copies the RTT, extending the genomic DNA strand with newly synthesized sequence containing the programmed nucleotide substitutions (red). (**b**) The newly synthesized edited strand forms a DNA flap intermediate (State 1). Flap processing and cellular DNA repair favor incorporation of the newly synthesized sequence (State 2), followed by resolution of the heteroduplex to generate edited DNA containing the programmed substitutions. Genomic DNA is shown in black, the continuous pegRNA in blue, newly reverse-transcribed DNA in purple/blue, programmed substitutions in red, and PAM sequences in green. Abbreviations: nCas9: Cas9 nickase; PAM: protospacer-adjacent motif; PBS: primer binding site; PE: prime editing; pegRNA: prime editing guide RNA; RT: reverse transcriptase; RTT: reverse transcription template.

Prime editing (PE) was introduced in 2019 as a “search-and-replace” genome editing strategy capable of directly writing new genetic information into a specified genomic site [1]. The original system combines a Cas9 H840A nickase (nCas9) fused to an engineered reverse transcriptase (RT) with a prime editing guide RNA (pegRNA). The pegRNA both directs the editor to its genomic target and contains a 3′ extension comprising a primer binding site (PBS) and reverse transcription template (RTT) encoding the intended sequence alteration. Following nicking of the target DNA strand, the exposed genomic 3′ end hybridizes to the PBS and primes reverse transcription of the RTT. Processing and resolution of the resulting DNA intermediates can then incorporate the programmed sequence into genomic DNA. The core molecular architecture and sequential steps of prime editing are illustrated in Figure 1. The original system enabled all 12 possible single-base substitutions as well as targeted insertions and deletions without requiring a DSB or exogenous donor DNA template [1].

The original study established the foundational PE1, PE2, PE3, and PE3b configurations [1]. Subsequent development has addressed multiple determinants of editing efficiency, fidelity, and product purity. Manipulation of cellular mismatch repair and optimization of editor architecture produced enhanced systems including PE4, PE5, and PEmax [2]. Stabilization of the 3′ extension of pegRNAs generated engineered pegRNAs (epegRNAs) that can substantially improve editing efficiency [3]. Protein engineering and phage-assisted evolution subsequently produced compact and more active prime editor variants, including the PE6 family [4]. More recently, artificial intelligence-guided redesign of evolved reverse transcriptases has extended this engineering strategy to PE8 [5]. Collectively, these advances demonstrate that PE is not a single fixed editor but a modular platform in which the Cas protein, RT, pegRNA, DNA repair environment, and delivery configuration can each be optimized.

The range of genomic alterations accessible to PE has expanded in parallel. Whereas the original platform was particularly suited to substitutions and relatively small insertions and deletions, paired pegRNA architectures enable larger and more complex genomic modifications. Twin prime editing (twinPE), for example, uses two pegRNAs targeting opposite DNA strands to generate complementary edited intermediates and enables programmable deletion, replacement, integration, and inversion of larger DNA sequences [6]. Subsequent approaches have further expanded the scale and complexity of sequence modification accessible through PE-related technologies. These capabilities are particularly relevant to precision genetic medicine because pathogenic variation encompasses not only single-nucleotide substitutions but also insertions, deletions, splice-altering variants, repeat-associated changes, and larger genomic alterations.

Despite this rapid technological development, editing efficiency and therapeutic benefit are not equivalent. PE performance varies substantially with genomic target, pegRNA configuration, editor architecture, cell type, and tissue. Therapeutic translation is further constrained by the size and multicomponent nature of prime editors and by the challenge of delivering editing components efficiently to clinically relevant cells. Moreover, high molecular editing efficiency does not necessarily demonstrate phenotypic or functional rescue. Therapeutic evaluation must therefore consider product purity, unintended editing outcomes, genomic integrity, durability, tissue distribution, and the fraction of corrected cells required to alter disease phenotype. Distinguishing technological proof of concept from meaningful functional rescue is consequently essential when assessing the translational maturity of PE.

The expanding PE design space has also stimulated computational approaches for pegRNA selection, prediction of editing performance, and optimization of editor components. More recently, machine learning and artificial intelligence approaches have been applied directly to editor design, as illustrated by AI-guided redesign of reverse transcriptases in PE8 [5]. In parallel, paired editor systems, alternative polymerase configurations, and other emerging approaches are extending precise genome editing toward increasingly complex sequence modifications. However, these technologies vary substantially in experimental maturity and should not be interpreted as representing equivalent levels of therapeutic readiness.

Accordingly, the objective of this systematic review was to map and synthesize the technological and translational development of prime editing, with emphasis on editor architecture, pegRNA and protein engineering, DNA repair modulation, delivery, therapeutic applications, computational approaches, and emerging technologies. Systematic searches of PubMed and Web of Science followed by screening and manual re-screening identified 294 studies meeting the final eligibility criteria. Because the literature is highly heterogeneous with respect to editor configuration, genomic target, experimental system, delivery method, and reported outcome, the evidence was evaluated using systematic mapping and qualitative synthesis rather than quantitative meta-analysis. Particular attention was given to distinguishing technological proof of concept from evidence of functional or therapeutic benefit and to identifying the major barriers separating experimental editing performance from clinical translation.

## 2. Materials and Methods

### 2.1. Review Design

This systematic review was conducted and reported in accordance with the Preferred Reporting Items for Systematic Reviews and Meta-Analyses (PRISMA) 2020 framework. The objective was to systematically identify, map, and qualitatively synthesize the development of prime editing technologies, including editor architecture, pegRNA and protein engineering, DNA repair modulation, delivery, therapeutic applications, computational approaches, and emerging technologies. Because the included literature was highly heterogeneous with respect to editor configuration, genomic target, experimental model, delivery strategy, and reported outcome, the review was designed as a systematic mapping and qualitative thematic synthesis rather than a quantitative meta-analysis. The completed PRISMA 2020 checklist is provided in Supplementary Table S5. No review protocol was prospectively registered.

### 2.2. Literature Search Strategy

PubMed and Web of Science were searched on 1 August 2026 using the search term “Prime Editing.” The intentionally broad search retrieved 1892 records from PubMed and 1912 records from Web of Science (3804 records before deduplication and preliminary eligibility filtering). Records were imported into EndNote for reference management and duplicate identification. During preliminary processing, records outside the prespecified scope of the review were removed, including preprints (including bioRxiv and medRxiv). Crop-, agricultural-, plant-, and bacterial-focused studies were outside the predefined scope of this review, which focused on mammalian systems and applications relevant to precision genetic medicine, and were therefore excluded. Following EndNote deduplication and these preliminary exclusions, 2241 records entered the PRISMA study selection workflow. The database searches, retrieval counts, preliminary eligibility criteria, and study selection audit trail are documented in Supplementary Table S1.

### 2.3. Reference Management and Deduplication

Records retrieved from PubMed and Web of Science were imported into EndNote and merged into a single reference library. Duplicate records were identified using EndNote duplicate detection functions followed by manual review of bibliographic information where necessary. Within the 2241-record PRISMA identification set, 898 duplicate records were removed, leaving 1343 unique records for title and abstract screening. Bibliographic information was retained throughout screening to permit identification of duplicate publications and correction of inconsistent database records.

### 2.4. Eligibility Criteria

Studies were eligible for inclusion when prime editing constituted a substantive component of the experimental, technological, computational, delivery, or therapeutic investigation. Eligible studies included development or evaluation of prime editor architectures; Cas, reverse transcriptase, or pegRNA engineering; DNA repair modulation; computational or AI-guided PE design; delivery of PE components; application of PE in cellular or animal models; therapeutic correction of disease-associated variants; and emerging PE-related approaches that experimentally extended the scale, geometry, or mechanism of programmable editing.

Records were excluded when they were reviews, meta-analyses, editorials, conference abstracts, corrections without substantive primary data, preprints, duplicate publications, or lacked an identifiable author. Studies focused primarily on conventional CRISPR–Cas editing, base editing, or other genome editing technologies were excluded when PE was mentioned only incidentally and was not experimentally or computationally investigated. Publications before 2019 were excluded because prime editing was introduced in 2019. Studies addressing broader CRISPR biology, Cas structure or function, molecular dynamics, disease biology, or other biological topics without a substantive PE component were also excluded.

### 2.5. Study Screening and Final Eligibility Audit

Following removal of 898 duplicates, 1343 unique records underwent title and abstract screening. Of these, 865 were excluded during initial screening, leaving 478 candidate records for detailed eligibility assessment. During revision of the review, all 478 candidate records were manually re-screened against the prespecified eligibility criteria to ensure that the final evidence set contained primary studies with a substantive PE focus.

**Figure 2:**
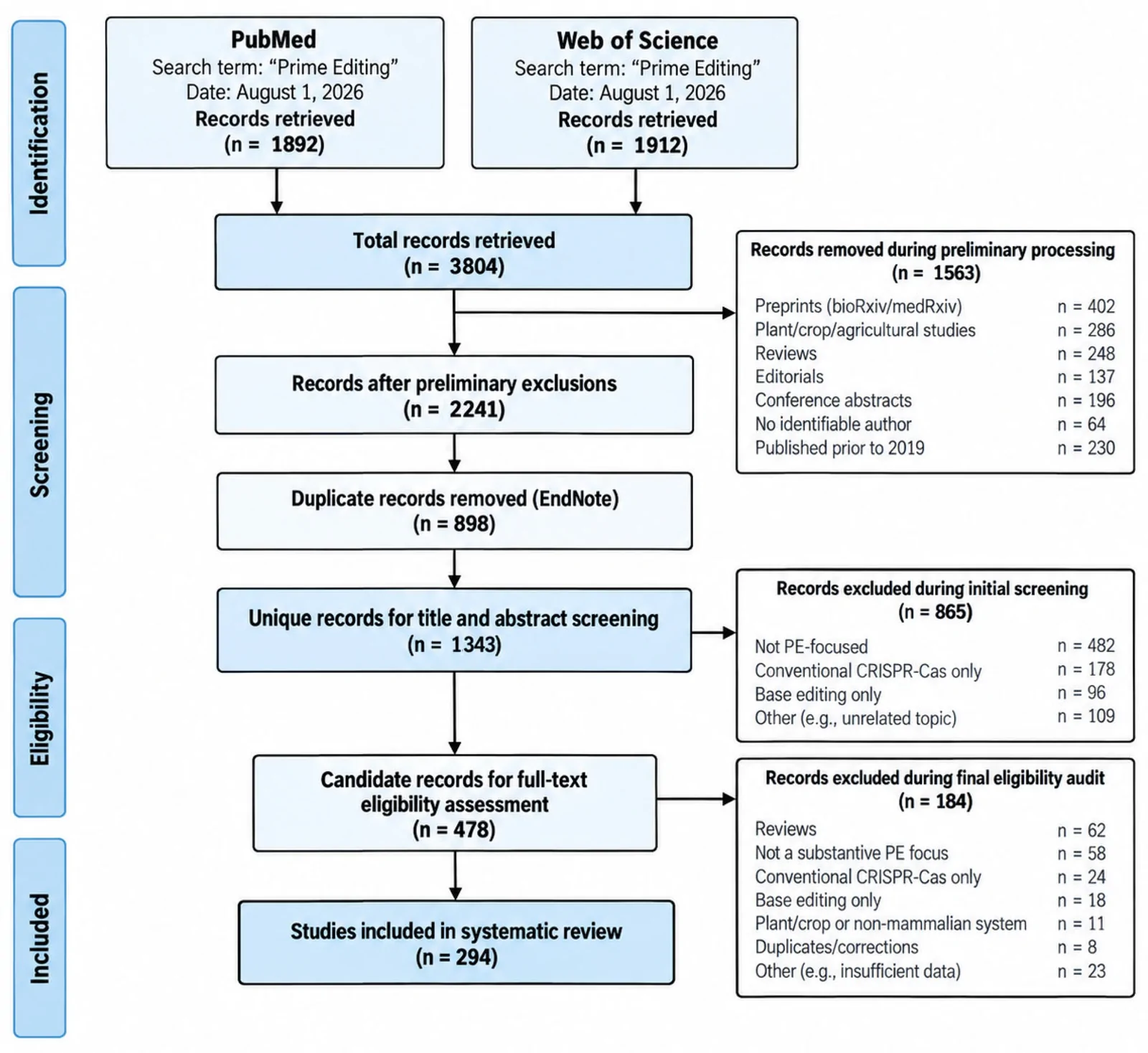
PRISMA 2020 flow diagram of study selection. PubMed and Web of Science were searched on 1 August 2026 using the term “Prime Editing,” yielding 1892 and 1912 records, respectively (3804 total). Following preliminary processing and deduplication, 1343 unique records underwent title and abstract screening. Initial screening excluded 865 records, leaving 478 candidate records for detailed eligibility assessment. All 478 candidate records were manually re-screened against the prespecified eligibility criteria; 184 were excluded during the final eligibility audit and 294 studies were retained in the systematic evidence synthesis. Detailed search information and study characteristics are provided in Supplementary Table S1, and the 184 final audit exclusions and reasons for exclusion are provided in Supplementary Table S3.

The final manual audit specifically reassessed records that could represent reviews, conventional CRISPR–Cas or base editing studies, studies in excluded plant or bacterial systems, corrections, duplicates, and studies in which PE was mentioned but was not a substantive component of the investigation. One hundred and eighty-four of the 478 candidate records were excluded during this final eligibility assessment, leaving 294 studies in the final systematic evidence synthesis. The study selection process is summarized in Figure 2, and all 184 records excluded during the final eligibility audit, together with their reasons for exclusion, are provided in Supplementary Table S3.

This revised flow preserves the distinction between the 865 records removed during initial screening and the 184 records removed during the final manual audit. The current manuscript already documents the 478-record manual re-screening and 294-study final evidence set.

### 2.6. Data Extraction and Evidence Classification

Data were extracted from the 294 studies retained in the final evidence set using a structured evidence matrix. Extracted information included publication characteristics, PE architecture (PE1, PE2, PE3, PE3b, PE4, PE5, PEmax, PE6, PE7, PE8, and twinPE), Cas and editor modifications, reverse transcriptase engineering, pegRNA design, DNA repair modulation, delivery modality, experimental setting or model, therapeutic application, computational or AI-guided approaches, and emerging or large-scale editing strategies.

Studies were classified into thematic categories including prime editing, reverse transcriptase, pegRNA, Cas modification, polymerase, delivery, therapeutics, AI/computational, and future/emerging technologies. These categories were non-mutually exclusive because individual studies could address multiple components of PE. Consequently, category counts and percentages were calculated relative to the 294-study evidence set and were not expected to sum to 100%.

For therapeutic studies, demonstration of sequence correction or molecular editing was distinguished from functional or phenotypic rescue, defined as experimentally demonstrated improvement in a disease-relevant molecular, cellular, physiological, or organism-level outcome. This distinction was used to avoid equating editing efficiency with therapeutic benefit.

Study-level characteristics and classifications are provided in Supplementary Table S1, detailed evidence extraction and study annotations in Supplementary Table S2, and the reference placement and evidence map in Supplementary Table S4. Records excluded during the final eligibility audit and their exclusion reasons are documented separately in Supplementary Table S3.

### 2.7. Evidence Synthesis

Substantial heterogeneity existed among studies with respect to editor architecture, genomic target, pegRNA configuration, experimental model, delivery method, reported editing metric, and biological outcome. Direct quantitative pooling of editing efficiencies across studies was therefore considered inappropriate, and no meta-analysis was performed. Instead, the 294 included studies were synthesized using systematic mapping and qualitative thematic analysis. The synthesis focused on the evolution of prime editor architectures; RT, pegRNA, and Cas engineering; DNA repair modulation; delivery strategies; therapeutic applications; computational and AI-assisted design; and experimentally demonstrated emerging technologies. Particular attention was given to the distinction between technological proof of concept and evidence of functional or therapeutic benefit and to the maturity and limitations of emerging approaches. Supplementary Table S2 provides the study-level evidence matrix, while Supplementary Table S4 maps included studies to the principal manuscript sections and evidence categories in which they are discussed.

### 2.8. Use of Generative Artificial Intelligence

Generative artificial intelligence (GenAI; ChatGPT, GPT-5.6 Sol, OpenAI; accessed September 2026) was used as an assistive tool during preparation and revision of this systematic review. GenAI assisted with organization and preliminary classification of the literature, preparation and refinement of study annotations and Supplementary Materials, editing and organization of selected manuscript text, and development of initial concepts and layouts for figures. Following identification of classification inconsistencies during manuscript review, the candidate evidence set was manually re-screened against the eligibility criteria, and the resulting study classifications and supplementary evidence tables were revised accordingly. GenAI-assisted outputs were reviewed, verified, and edited by the author. Final decisions regarding study inclusion and exclusion, evidence classification, interpretation of the literature, scientific conclusions, and manuscript content were made by the author. GenAI was not used to generate experimental data or primary research results. The author takes responsibility for the content of the review.

## 3. Results

The systematic searches identified 2241 records, of which 898 were removed as duplicates. Initial screening of the remaining 1343 unique records excluded 865 records and yielded 478 candidate studies for detailed eligibility assessment. Manual re-screening of all 478 candidate records against the final eligibility criteria excluded an additional 184 records, leaving 294 studies in the final systematic evidence synthesis (Figure 2). Characteristics and study-level classifications of the included evidence are provided in Supplementary Tables S1 and S2, the 184 studies excluded during the final eligibility audit and their reasons for exclusion are documented in Supplementary Table S3, and Supplementary Table S4 maps the included studies to the principal manuscript sections and provides the corresponding EndNote citations.

The evidence base was highly heterogeneous, encompassing development and optimization of prime editor architectures, pegRNA and reverse transcriptase (RT) engineering, Cas protein modification, DNA repair modulation, delivery technologies, computational approaches, and therapeutic applications. Because many studies addressed more than one aspect of PE, the classifications were non-mutually exclusive; consequently, category counts and percentages are not expected to sum to the total number of included studies or to 100%.

### 3.1. Evidence Landscape and Technological Development

Prime editing research expanded rapidly following the introduction of the technology in 2019 [1]. Among the 294 studies included in the final systematic evidence synthesis, one was published in 2019, followed by five in 2020, 26 in 2021, 44 in 2022, 47 in 2023, 44 in 2024, 74 in 2025, and 53 through the 11 August 2026 search date. Thus, 127 studies (43.2%) were published in 2025 or during the first eight months of 2026, demonstrating that a substantial proportion of the evidence base is recent.

The dominant classification was prime editing technology or methodology, represented by 272 studies (92.5%). Within the overlapping thematic classifications, 90 studies (30.6%) addressed therapeutic applications, 79 (26.9%) addressed pegRNA design or optimization, 51 (17.3%) investigated delivery, and 45 (15.3%) addressed Cas modification. Smaller but increasingly important components included 15 studies (5.1%) classified as computational or AI-related, 14 (4.8%) addressing reverse transcriptase (RT) engineering, five (1.7%) classified as future or emerging technologies, and two (0.7%) addressing DNA polymerase-based approaches. These classifications were non-mutually exclusive because individual studies frequently addressed multiple components of PE; consequently, the counts and percentages should not be summed.

Methodological expansion has been accompanied by increasing attention to product quality and genomic safety. Studies have examined pegRNA-independent off-target effects [7], developed PE-specific or broadly applicable approaches for identifying off-target editing [8,9,10], and investigated potential epigenetic alterations associated with PE [11]. This literature reinforces the distinction between maximizing the percentage of intended editing and optimizing the overall editing outcome, which additionally requires consideration of unintended products, off-target activity, and genomic integrity.

PE has also expanded across increasingly diverse experimental settings. Optimized systems have been applied in human iPSCs [12,13], mouse embryos [14], mice and rabbits [15], primary neurons [16], and other experimental models. Delivery has progressed in parallel from readily transfected cultured cells toward viral, nonviral, RNP, ex vivo, and in vivo systems, including dual-AAV editing in differentiated mouse brain, liver, and heart [17]. These advances are translationally important because improvements measured under optimized cell culture conditions do not necessarily persist when the complete editing system must be delivered to a disease-relevant tissue.

**Figure 3:**
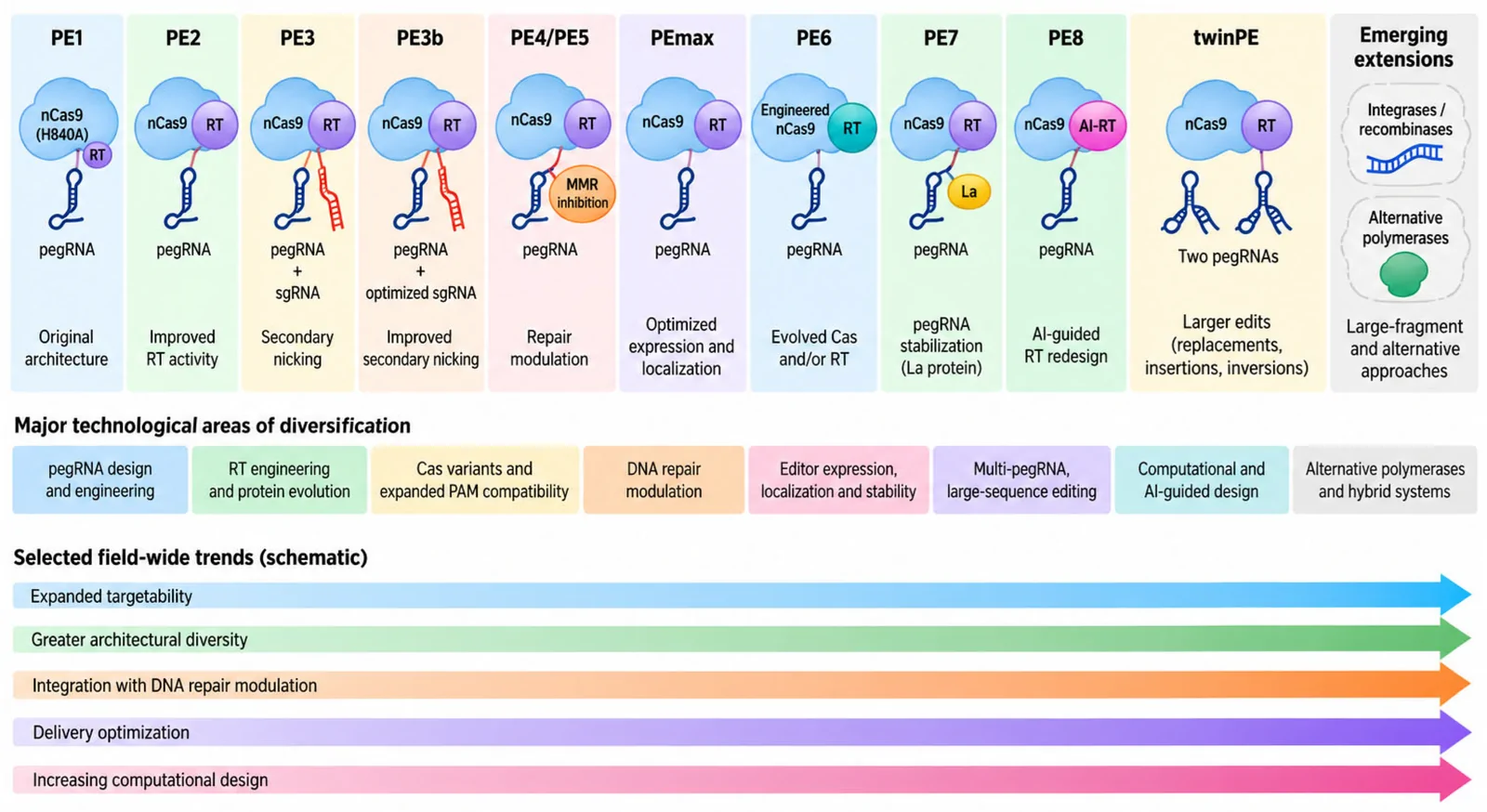
Evolution and diversification of prime editing architectures. Major PE architectures have diversified through RT and pegRNA engineering, Cas modification, DNA repair modulation, and computational design. PE1–PE3b established the core architecture and secondary strand nicking [1]; PE4/PE5 and PEmax incorporated repair and editor optimization [2]; PE6–PE8 introduced evolved proteins, pegRNA stabilization, and AI-guided RT redesign [4,5,18]; and twinPE expanded programmable edit size [6]. Emerging hybrid systems further extend large-fragment editing. Arrows indicate broad technological trends and do not imply universal superiority of later PE systems. Abbreviations: AI: artificial intelligence; MMR: mismatch repair; PE: prime editing; pegRNA: prime editing guide RNA; RT: reverse transcriptase; twinPE: twin prime editing.

Collectively, the 294-study evidence set depicts PE as an increasingly modular genome editing platform rather than a single fixed editor. Cas proteins determine target recognition and nick geometry; RTs influence templated DNA synthesis; pegRNAs provide targeting and sequence information; cellular repair pathways influence resolution of editing intermediates; delivery systems determine whether these components reach the relevant cells; and computational methods increasingly assist selection or engineering of these components. The major architectural transitions underlying this diversification are examined in Section 3.2 and summarized in Figure 3.

### 3.2. Evolution of Prime Editor Architectures

The evolution of PE has involved modification of nearly every component of the original system, including the RT, Cas protein, pegRNA, secondary strand nicking strategy, and cellular DNA repair environment (Figure 3). The resulting systems are best viewed as complementary architectural branches rather than a simple linear sequence in which each newer PE generation supersedes its predecessors. Performance remains strongly dependent on target sequence, intended edit, pegRNA design, cellular context, desired product purity, and delivery constraints.

The original PE architecture established the basic mechanistic framework for subsequent development. PE1 combined an H840A Cas9 nickase with Moloney murine leukemia virus RT and a pegRNA containing a spacer, primer binding site (PBS), and reverse transcription template (RTT). PE2 incorporated an engineered RT to increase editing activity. PE3 added a second guide RNA that nicks the non-edited strand and can increase intended editing but may also increase unwanted indels, whereas PE3b positions the secondary nick so that it preferentially occurs after installation of the intended edit [1]. Thus, PE3 and PE3b established an early architectural tradeoff between editing efficiency and product purity, rather than simply representing progressively more active versions of the same editor.

Subsequent approaches demonstrated that PE performance could be improved without fundamentally changing the Cas–RT catalytic architecture. Addition of the Rad51 DNA binding domain to PE2 increased editing in multiple contexts, demonstrating that recruitment of DNA-interacting domains could modify editor performance [19]. Bidirectional priming provided another architectural strategy for increasing productive editing [20], while engineered pegRNA expression systems broadened the ways in which the RNA component could be generated and used [21]. Structural stabilization of pegRNAs likewise improved PE through modification of the RNA substrate rather than the catalytic protein [3,22,23]. These studies established early that PE efficiency is an emergent property of the complete editor–pegRNA–target system.

A second major architectural transition came from recognition that cellular DNA repair is an active determinant of PE outcome. CRISPR interference screens identified MMR as a major barrier to productive PE and showed that MMR can promote unwanted editing products. Transient MMR inhibition using dominant negative MLH1 generated PE4 and PE5, corresponding broadly to PE2- and PE3-like editing in an MMR-suppressed environment, while additional protein and expression optimization produced PEmax [2]. This represented an important conceptual shift: optimization no longer focused solely on the editing enzyme but also on controlling the cellular pathways that resolve the editing intermediate.

The importance of cellular context has subsequently been demonstrated in multiple settings. Transient MMR inhibition substantially improved precise editing in early mouse embryos [14], while modulation of methylation on newly synthesized DNA and prolonged editor expression provided another strategy for increasing productive PE [24]. AI-designed inhibition of MLH1 has more recently provided an alternative route to manipulating the same biological bottleneck [25]. Together, these studies indicate that repair modulation constitutes an architectural dimension of PE rather than merely an experimental accessory.

Modification of nickase and nuclease properties provides another distinct branch. Genuine Cas9 nickases have been used to reduce unwanted indel formation during PE [26], whereas nuclease-based prime editors have explored the tradeoff between increased DNA cleavage and precise sequence installation [27]. These approaches emphasize that Cas engineering affects not only which genomic sites can be targeted but also the DNA intermediates presented to cellular repair pathways and therefore the distribution of intended and unintended products.

Targeting range has expanded in parallel through modification or replacement of the Cas component. PAM-flexible Cas9 variants broaden the number of genomic positions accessible to PE [28], whereas alternative CRISPR proteins, including Cas12a-based PE architectures, provide distinct PAM requirements and targeting geometries [29]. This expansion is particularly important for precision correction of pathogenic variants because PE generally requires appropriate spatial positioning of the nick relative to the intended sequence alteration. Increasing PAM flexibility can therefore make previously inaccessible variants editable, although broader targeting must be considered together with specificity and product purity.

The RNA component has likewise become increasingly integrated into editor architecture. Engineered pegRNAs stabilize the vulnerable 3′ PBS-RTT extension and can substantially improve productive editing [3]. Additional structural approaches have altered pegRNA sequence or RNA motifs to increase stability or editing performance [22,23]. Recruitment of the endogenous small RNA-binding protein La subsequently demonstrated that cellular RNA-binding proteins can be exploited to stabilize pegRNAs and enhance PE, leading to the PE7 architecture [18]. PE7 therefore represents a different optimization strategy from PE4–PE6: rather than primarily modifying DNA repair or catalytic activity, it increases productive use of the RNA component.

Protein evolution generated another major architectural branch. Phage-assisted evolution and protein engineering produced the PE6 family, including compact and more active RT and Cas variants designed for different editing contexts [4]. These studies showed that RT properties interact with RTT length, structure, and edit type, arguing against the existence of a single universally optimal RT. More recently, structure-informed AI-guided redesign of laboratory-evolved RTs generated the PE8 family [5]. PE8 extends editor engineering from rational mutation and laboratory evolution toward computational protein redesign, but its reported improvements remain target and context dependent and should not be interpreted as universal superiority over earlier PE architectures.

Architectural diversification has also expanded the scale and geometry of editing. Paired PE strategies demonstrated that coordinated editing at two sites could generate targeted genomic translocations and inversions [30].

Twin prime editing (twinPE) extended this principle by using two pegRNAs targeting opposite strands to generate complementary edited intermediates, enabling programmable deletion, replacement, integration, and inversion of larger DNA sequences [6]. These approaches are mechanistically distinct from simply increasing the efficiency of PE2 or PE3 because they change the number and spatial organization of editing reactions required to construct the final product.

Several studies further illustrate that architectural optimization is often cell- and application-specific. PEmax, epegRNAs, and repair modulation have been combined to generate isogenic human iPSC models [12]; optimized PE systems have been developed for mice and rabbits [15]; PE3b has been adapted for primary neurons [16]; and all-in-one inducible architectures have been developed for human pluripotent stem cells [13]. These findings caution against ranking PE architectures using efficiency measurements obtained in a single cell line or genomic target.

The expanding architecture space has also stimulated efforts to improve activity through additional cellular or molecular interactions. Recruitment of the pioneer transcription factor P65 enhanced PE in selected contexts [31], exonuclease-enhanced prime editors provided another route to increasing productive editing [32], and anti-CRISPR protein modulation has recently been investigated as a means of altering PE activity and product profiles [33]. These systems reinforce the broader conclusion that PE development is no longer adequately described by a single PE1-to-PE8 lineage.

Collectively, the evidence supports a multidimensional rather than linear model of prime editor evolution. The optimal architecture depends on the intended edit, genomic context, desired product purity, cell type, delivery requirements, and biological objective. Figure 3 summarizes these complementary architectural branches without implying universal superiority of later PE systems. The major PE architectures and emerging extensions are compared in Table 1, emphasizing their mechanistic differences, editing capabilities, translational considerations, and current evidence maturity.

### 3.3. pegRNA and Reverse Transcriptase Engineering

Engineering of the prime editing guide RNA (pegRNA) and reverse transcriptase (RT) represents a major axis of PE optimization. In the final evidence set, 79 of 294 studies (26.9%) addressed pegRNA design or optimization and 14 (4.8%) addressed RT engineering. These categories were non-mutually exclusive because the pegRNA template, RT, Cas protein, target sequence, and cellular environment function as an integrated editing system. The evidence increasingly indicates that PE performance cannot be optimized by considering either the pegRNA or RT in isolation.

The pegRNA performs two essential functions: its spacer directs the editor to the genomic target, whereas its 3′ extension contains the primer binding site (PBS) and reverse transcription template (RTT) that initiate reverse transcription and encode the intended sequence alteration [1]. Consequently, PE efficiency depends not only on target recognition but also on PBS annealing, RTT composition, RNA secondary structure, accessibility, and stability. Early computational design tools formalized selection of these parameters and facilitated systematic evaluation of candidate pegRNAs [41,42,43].

Early experimental optimization demonstrated that pegRNA processing and stability are major determinants of editing efficiency. Csy4-mediated processing of pegRNAs increased PE activity, illustrating that the manner in which the guide RNA is generated can affect productive editing [44]. RNA structural engineering subsequently provided several complementary strategies for stabilizing the vulnerable 3′ extension. Incorporation of RNA G-quadruplex structures enhanced PE efficiency [45], whereas addition of an exoribonuclease-resistant RNA (xrRNA) motif similarly improved pegRNA performance [23]. These studies established that the pegRNA is not simply an informational template but an engineerable structural component of the PE reaction.

A major advance was the development of engineered pegRNAs (epegRNAs). Degradation of the pegRNA 3′ extension can remove portions of the PBS and RTT and thereby reduce productive editing. Nelson et al. addressed this limitation by incorporating structured RNA motifs at the pegRNA 3′ terminus, protecting the extension from degradation and substantially increasing editing efficiency across multiple human cell lines and primary fibroblasts [3]. The study also introduced pegLIT to assist selection of linkers between the pegRNA and stabilizing structural motif. Together with other structural stabilization strategies [23,45], these findings established 3′ extension stability as an experimentally tractable determinant of PE performance.

PBS and RTT configuration provide another major level of pegRNA optimization. PBS length and sequence affect hybridization of the nicked genomic 3′ end, whereas RTT length, composition, and secondary structure influence synthesis and incorporation of the edited DNA intermediate. Importantly, the evidence does not support a single universally optimal PBS or RTT configuration. Intramolecular complementarity between the PBS and spacer can itself inhibit productive editing, and disrupting this complementarity can improve PE efficiency [46]. Loop engineering provides another strategy for altering pegRNA structure and increasing productive editing [46], while mismatch-containing pegRNAs have been developed to improve intended editing and reduce indel formation in evaluated systems [47]. These studies demonstrate that PBS and RTT optimization is fundamentally target-dependent, reflecting interactions among sequence complementarity, RNA folding, genomic sequence, and editor architecture.

Importantly, pegRNA engineering can influence product quality as well as editing efficiency. Modified pegRNAs have been designed specifically to mitigate scaffold-derived PE byproducts [48], whereas mismatch-containing pegRNAs can alter the balance between intended editing and indel formation [47]. These findings reinforce a recurring theme of the systematic evidence synthesis: optimization should not be defined solely by the percentage of alleles containing the intended edit. Product purity and unintended editing products are additional measures of performance.

The RNA design space has continued to diversify beyond conventional linear pegRNAs. Adaptable template RNA architectures have been developed to expand the editable space of nuclease-based prime editors [49], while circular RNA-mediated inverse PE enables editing in an alternative orientation relative to the nick site [50]. These approaches illustrate that RNA engineering can modify not only efficiency but also editing geometry and accessible sequence space.

The importance of pegRNA stability is further demonstrated by interactions with endogenous cellular proteins. Genome-scale CRISPR interference screening identified the small RNA-binding protein La as a positive determinant of PE activity. La can stabilize appropriately configured pegRNAs and enhance editing across multiple PE architectures, edit classes, loci, and cell types [18]. Incorporation of the RNA-binding N-terminal domain of La into the editor generated PE7, which improved editing with conventional pegRNAs, epegRNAs, and synthetic pegRNAs optimized for La binding. This work established a distinct strategy in which productive editing is increased by manipulating cellular recognition and stabilization of the pegRNA, rather than modifying the RT catalytic reaction itself.

The growing diversity of pegRNA architectures has also enabled increasingly complex experimental applications. High-efficiency pegRNA/epegRNA platforms have supported multiplexed PE screening [51], successive rounds of marker-free editing [52], and multiplexed editing in additional experimental systems [53]. These studies demonstrate that pegRNA engineering influences not only the efficiency of individual edits but also the scalability and experimental utility of PE.

RT engineering addresses the complementary enzymatic component of the reaction. The original PE1 architecture used wild-type Moloney murine leukemia virus RT, whereas PE2 incorporated an engineered M-MLV RT with mutations that increased editing activity [1]. This transition established early in the development of PE that the biochemical properties of the polymerase copying the RTT could substantially affect editing outcome. Subsequent optimization in diverse experimental systems has continued to combine RT modification with changes in pegRNA and editor architecture [54].

Structural studies have provided a mechanistic basis for the functional coupling between pegRNA and RT. Structural characterization of the PE complex revealed how the Cas9–RT architecture accommodates pegRNA-guided reverse transcription and provided direct insight into interactions among the nicked DNA substrate, pegRNA, and RT during synthesis of the edited strand [55]. These findings provide structural support for an important conclusion from the broader evidence base: RT performance is inherently dependent on the architecture and conformation of the RNA and DNA substrates presented to it.

A major advance in RT engineering came from protein engineering and phage-assisted evolution. These approaches generated the PE6 family, including compact and more active RT and Cas variants optimized for different editing contexts [4]. The PE6 studies demonstrated that different RTs can perform preferentially with different edit types, RTT lengths, and RNA structures. Compact RTs additionally reduce overall editor size, linking catalytic optimization to the delivery constraints discussed in Section 3.5. PE6 therefore illustrates why RT engineering must consider activity, substrate compatibility, protein size, and delivery rather than catalytic activity alone.

More recent approaches have combined rational and computational design with RT and editor optimization. Rational design together with AI-driven optimization has been used to alter editing geometry and improve fidelity in evaluated systems [56]. Most notably, Tao et al. used structure-informed AI to redesign laboratory-evolved RTs after identifying a tradeoff in which some mutations associated with increased editing activity reduced protein stability and expression [5]. The resulting PE8 family was designed to improve the balance among RT activity, protein stability, expression, and editing performance. PE8 therefore extends RT optimization from rational engineering and laboratory evolution toward AI-guided protein redesign.

The increasing integration of RNA and protein engineering argues against treating pegRNA and RT optimization as independent technological tracks. Changes in PBS length, RTT sequence, RNA secondary structure, and pegRNA stabilization alter the substrate encountered by the RT, whereas changes in RT activity, processivity, stability, and structure can alter which pegRNA configurations perform optimally. Structural analysis [55], evolved RTs in PE6 [4], and AI-guided RT redesign in PE8 [5] collectively reinforce this mechanistic interdependence.

**Figure 4:**
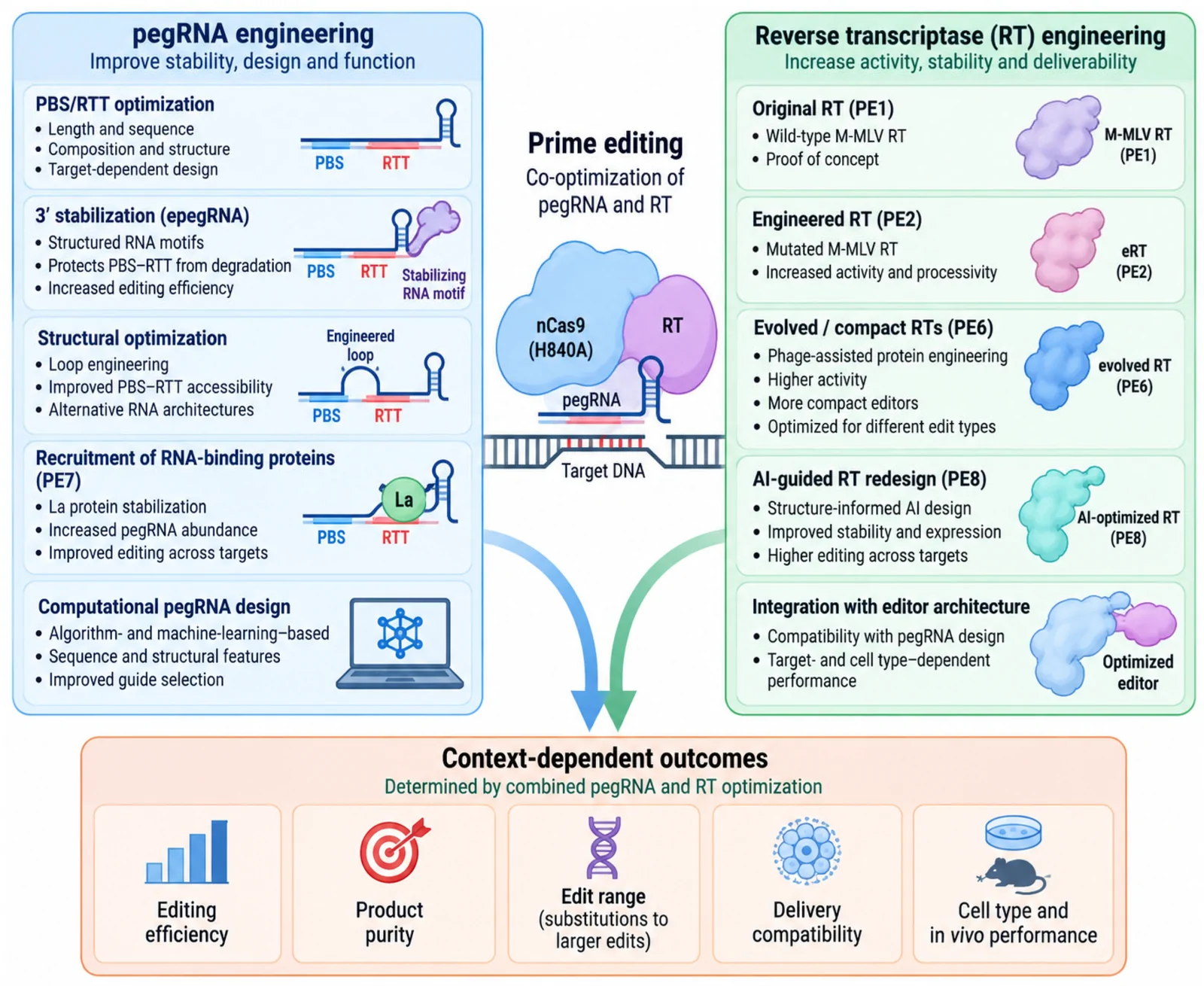
Complementary pegRNA and reverse transcriptase engineering strategies for prime editing optimization. pegRNA engineering improves PBS/RTT design, RNA stability and accessibility, including epegRNAs and La-mediated stabilization in PE7 [3,18]. RT engineering progressed from the original and engineered M-MLV RTs to evolved PE6 variants and AI-guided PE8 redesign [1,4,5]. These complementary strategies converge on context-dependent effects on editing efficiency, product purity, edit range, delivery compatibility, and cellular or in vivo performance. Abbreviations: AI: artificial intelligence; PBS: primer binding site; PE: prime editing; pegRNA: prime editing guide RNA; RT: reverse transcriptase; RTT: reverse transcription template.

Overall, the evidence demonstrates a progression from optimization of individual pegRNA sequence parameters toward co-optimization of RNA structure, RNA stability, RT properties, and editor architecture. Early pegRNA design tools [41,42,43], RNA processing and stabilization strategies [3,23,44,45], PBS/RTT and structural engineering [46,47,57], cellular stabilization in PE7 [18], evolved RTs in PE6 [4], and AI-guided RT redesign in PE8 [5] demonstrate that PE performance emerges from interactions among the RNA template, RT, genomic target, and cellular environment. Accordingly, no particular pegRNA configuration or RT architecture should be considered universally optimal, and improvements in editing efficiency should be evaluated together with product purity, specificity, delivery compatibility, and biological outcome. These complementary routes to optimization are summarized in Figure 4.

### 3.4. Cas Engineering and DNA Repair Modulation

Cas engineering and manipulation of cellular DNA repair pathways represent complementary strategies for expanding the targeting range of prime editing and improving the efficiency and purity of editing outcomes. In the final evidence set, 45 of 294 studies (15.3%) addressed Cas protein modification. These studies included altered PAM recognition, alternative Cas proteins, modified nickases, and other changes to the targeting component. In parallel, mechanistic studies demonstrated that endogenous DNA repair pathways actively determine whether prime editing intermediates are retained, rejected, or converted into unintended products. Together, these findings support a model in which editing outcome is determined jointly by where and how the Cas protein initiates editing and how the cell subsequently resolves the resulting DNA intermediate.

A major limitation of conventional SpCas9-based prime editing is the requirement for an appropriately positioned protospacer-adjacent motif (PAM). Precise correction can impose stricter geometric constraints than gene disruption because the nick, primer binding site (PBS), reverse transcription template (RTT), and intended alteration must be appropriately positioned relative to one another. PAM-flexible prime editors therefore broaden not only the number of targetable sequences but also the possible editing geometries. Engineered SpCas9 variants demonstrated that altered PAM recognition could substantially expand the targeting scope of PE [28]. The corrected evidence map specifically classifies this study as both Cas modification and PAM-flexible prime editing.

Expansion beyond conventional SpCas9 provides another route to increasing target accessibility. Cas12a-based prime editors coupled to circular RNA designs were developed to exploit a smaller Cas protein, T-rich PAM recognition, and the potential for multiplex targeting [29]. Nickase- and nuclease-dependent Cas12a prime editors achieved editing in human cells and demonstrated multiplex editing of several genomic targets. Together with PAM-relaxed SpCas9 variants, these results demonstrate that the Cas component of PE is increasingly an interchangeable targeting module rather than a fixed element of the original architecture.

Cas engineering can also improve product purity rather than simply expand target range. The H840A Cas9 nickase used in conventional PE retains low-level capacity to generate double-strand breaks under some conditions. Additional modification of the HNH domain generated “genuine” Cas9 nickases with reduced double-strand cleavage activity. When incorporated into PE2 and PE3, these variants reduced unwanted indels, and selected configurations increased the proportion of correctly edited products [26]. Earlier comparisons of nickase- and nuclease-based PE likewise demonstrated that altering Cas cleavage activity changes the balance among editing efficiency, intended products, and undesired outcomes [58]. These studies emphasize that maximal Cas activity is not necessarily optimal for precision editing.

The importance of Cas cleavage behavior is particularly evident in nuclease-based prime editing. Replacement of the nickase with an active Cas9 nuclease can improve editing at sites that respond poorly to conventional PE but increases the complexity of repair products. Subsequent optimization of nuclease prime editors demonstrated that manipulation of DNA repair can improve precision, further illustrating that Cas architecture and cellular repair cannot be considered independently [27]. Thus, the systematic evidence supports a continuum of Cas architectures in which increased cleavage activity may improve editing at difficult targets but can introduce a corresponding requirement for greater control over DNA repair outcomes.

The central role of endogenous repair was established particularly clearly by studies of DNA mismatch repair (MMR). Prime editing generates heteroduplex intermediates in which the newly synthesized edited strand can contain mismatches with the original genomic strand. MMR can recognize these intermediates and oppose retention of the intended sequence. Inhibition of MMR using dominant negative MLH1 (MLH1dn) substantially increased editing in MMR-proficient cells and contributed to development of PE4 and PE5, which combine MMR inhibition with PE2- and PE3-like architectures, respectively [2]. This work established DNA repair as an experimentally controllable component of the prime editing system rather than merely a source of target-to-target variability.

More recent work has refined this approach. Rather than relying exclusively on the relatively large MLH1dn protein, Park et al. used generative protein design methods to produce a compact MLH1 small binder (MLH1-SB) that interferes with the MLH1–PMS2 interaction [25]. Integration of this repair-modulating component into PE architectures produced the PE-SB platform and increased editing in cultured cells and in vivo. The result is important conceptually because it demonstrates that DNA repair modulation itself can be engineered as a modular component of PE and, increasingly, can be optimized computationally.

The influence of MMR is also strongly dependent on cellular context. In human pluripotent stem cells (hPSCs), combined modulation of the DNA damage response and repair pathways improved precise genome editing, including enhancement of PE through dominant negative MLH1 together with inhibition of p53-dependent responses [59]. These findings reinforce a broader conclusion of the evidence synthesis: a PE configuration optimized in one cell type cannot necessarily be assumed to perform similarly in another, because DNA repair capacity and DNA damage responses vary among cellular systems.

Repair modulation has also expanded beyond MMR. Dacquay et al. demonstrated that combined pharmacological inhibition of DNA-dependent protein kinase (DNA-PK) and DNA polymerase theta (Polθ) improved the precision of multiple PE architectures, including PE3, PE5, PE7 and paired pegRNA systems [60]. The intervention reduced both PE-independent indels and PE-associated byproducts such as template duplications, indicating that classical and alternative end-joining pathways contribute to unwanted resolution of editing intermediates. The study therefore extends repair engineering beyond MMR and demonstrates that several competing DNA repair pathways can influence PE product distributions. This conclusion is consistent with the broader mechanistic literature identifying MMR and end-joining pathways as important determinants of PE outcome.

Secondary strand nicking provides another illustration of the interaction between editor architecture and repair. PE3 increases editing by nicking the non-edited strand and thereby biasing resolution toward the newly synthesized edited strand, but this strategy can also increase indel formation. PE3b reduces this liability by positioning the second nick so that it preferentially occurs after successful installation of the intended sequence [1]. Thus, the difference between PE3 and PE3b is fundamentally a repair-biasing strategy, demonstrating that the timing and geometry of Cas-mediated nicking can be used to influence cellular resolution of the edited intermediate.

Collectively, these studies reveal a recurring tradeoff among targetability, editing efficiency, and product purity. PAM-flexible editors increase the number and geometry of accessible targets [28]; alternative Cas architectures further diversify targeting [29]; genuine nickases can reduce unwanted indels [26]; MMR modulation increases productive editing [2,25]; and inhibition of competing end-joining pathways can improve product precision [60]. However, greater molecular editing activity does not by itself establish a superior therapeutic editor. Repair pathways such as MMR, DNA-PK, and Polθ contribute to normal genome maintenance, making the duration and cellular specificity of their manipulation important considerations for translation.

**Figure 5:**
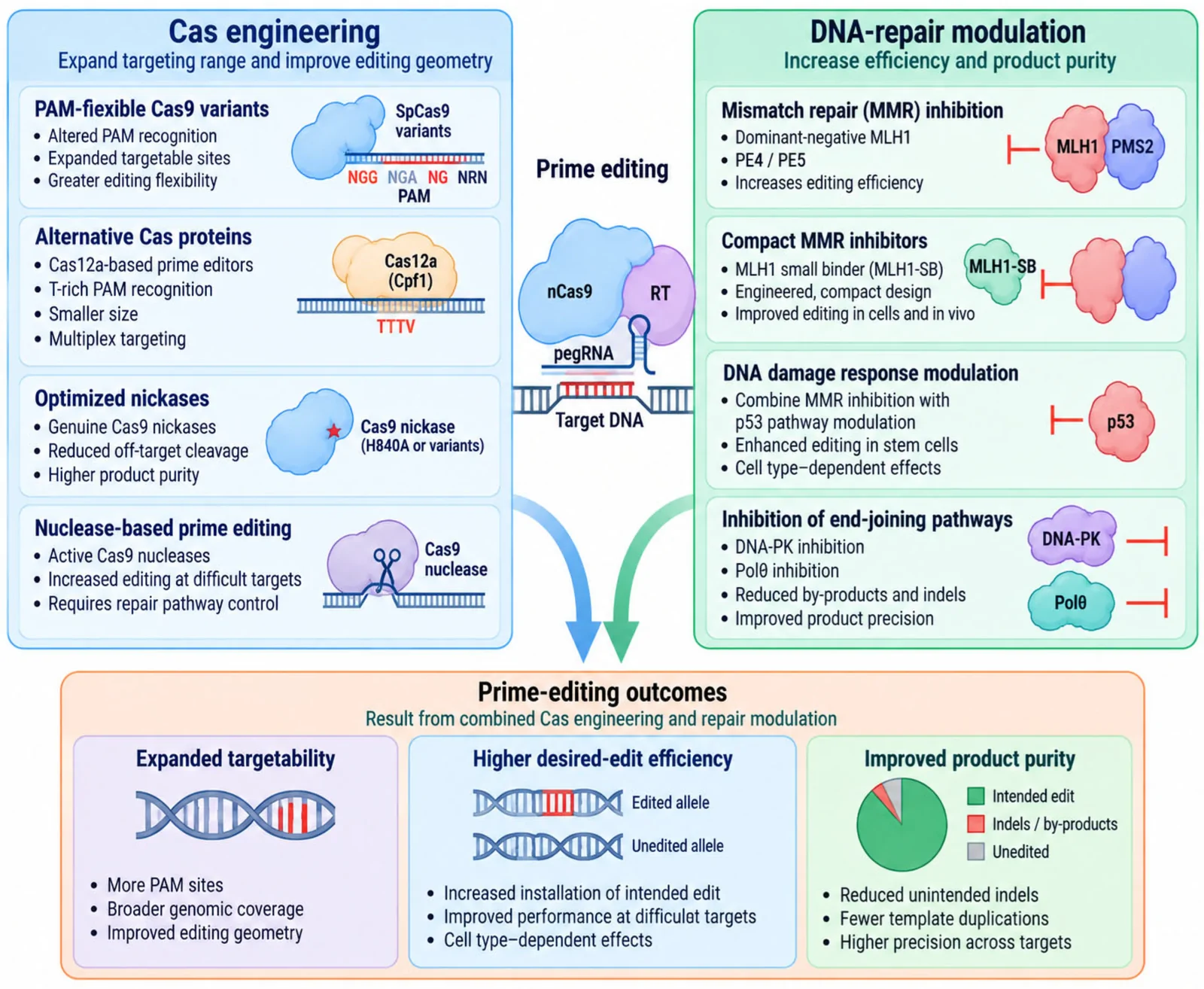
Cas engineering and DNA repair modulation as complementary determinants of prime editing outcomes. Cas engineering expands target accessibility through PAM-flexible Cas9 variants, alternative Cas proteins such as Cas12a, optimized nickases, and nuclease-based architectures [26,28,29,58]. DNA repair modulation acts downstream of editing initiation: inhibition of mismatch repair underlies PE4/PE5 [2], whereas compact MLH1 inhibitors and modulation of additional DNA damage and end-joining pathways can further alter editing efficiency and product purity [25,59,60]. The two strategies converge on three principal outcomes—targetability, desired edit efficiency, and product purity—and improvements in one parameter do not necessarily imply improvement in the others. Abbreviations: Cas: CRISPR-associated protein; DNA-PK: DNA-dependent protein kinase; MMR: mismatch repair; MLH1: MutL homolog 1; PAM: protospacer-adjacent motif; PE: prime editing; Polθ: DNA polymerase theta; RT: reverse transcriptase.

The evidence therefore supports two interacting levels of optimization: Cas engineering determines target accessibility and the nature of the initiating DNA lesion, whereas repair engineering influences the resolution of the resulting editing intermediate. Neither can be optimized independently of the other. These relationships, and their convergence on targetability, editing efficiency, and product purity, are summarized in Figure 5.

### 3.5. Delivery Strategies

Efficient delivery remains one of the principal barriers separating prime editing from broad therapeutic application. In the final evidence set, 51 of 294 studies (17.3%) addressed delivery. Prime editors present an unusually demanding delivery problem because the active system may require a large Cas–reverse transcriptase (RT) fusion, one or more prime editing guide RNAs (pegRNAs), and, depending on the architecture, an additional nicking guide, repair-modulating component, or second pegRNA. Consequently, improvements in molecular editing efficiency do not necessarily translate into effective in vivo editing if all required components cannot reach the appropriate cells at sufficient levels and stoichiometry.

Adeno-associated virus (AAV) has been one of the principal platforms for in vivo PE, but the size of conventional Cas9–RT prime editors exceeds the packaging capacity of a single AAV vector. Early in vivo studies demonstrated that AAV-delivered prime editors could introduce targeted sequence changes in adult mouse tissues, including pathogenic allele correction and cancer modeling [61]. This packaging constraint subsequently stimulated development of split editor architectures. A flexible split prime editor incorporating a truncated RT improved dual-AAV delivery and enabled in vivo editing in mouse liver [62], whereas an alternative split architecture separated the RT from the Cas targeting component and used an untethered RT together with a circular RNA template [63]. These studies established that the original covalently linked Cas9–RT architecture can be reorganized to accommodate delivery constraints.

Subsequent optimization substantially improved dual-AAV PE in vivo. Davis et al. systematically addressed several delivery bottlenecks and demonstrated efficient PE in mouse brain, liver, and heart using dual-AAV systems [17]. These experiments are important because they demonstrate substantial editing in differentiated tissues rather than only cultured cells, while also illustrating the strong dependence of editing performance on tissue and target. Nevertheless, dual-vector delivery requires successful uptake and expression of multiple components within the same cell and therefore introduces an additional constraint compared with single-vector systems.

Reducing editor size provides a complementary solution to viral packaging limitations. Protein engineering and phage-assisted evolution generated the PE6 family, including compact RT-containing editors designed to retain or improve editing activity while reducing overall editor size [4]. Smaller Cas proteins provide another route toward delivery-compatible architectures. Engineering of CjCas9, for example, has been explored for PE in part because its reduced size is more compatible with AAV packaging [64]. These findings illustrate an increasingly important principle: editor engineering and delivery engineering are interdependent, because gains in catalytic activity or editing scope have limited translational value if the resulting editor cannot be efficiently packaged and delivered.

Although viral vectors provide efficient tissue transduction, prolonged intracellular expression of a genome editor may not always be desirable. Transient delivery therefore represents an important alternative. Engineered virus-like particles (eVLPs) can package prime editor ribonucleoprotein (RNP) complexes rather than DNA encoding the editor, thereby providing a transient, transgene-free delivery strategy. An et al. demonstrated eVLP-mediated delivery of prime editor RNP complexes in vivo [65]. More recent work has extended eVLP-mediated PE toward compound delivery and high-throughput genome engineering applications [66]. Such approaches potentially reduce the duration of editor exposure while avoiding the requirement for intracellular transcription and translation of the complete PE protein.

RNA- and RNP-based delivery provides a related strategy for transient editing. Delivery of prime editor protein or mRNA together with pegRNA avoids sustained expression of editor-encoding DNA, but it introduces challenges involving RNA stability, manufacturing, formulation, and coordinated intracellular delivery. These constraints are particularly important for pegRNAs because they are substantially longer and structurally more complex than conventional single-guide RNAs. Methods for generating long, chemically modified pegRNAs have improved the practicality of RNP- and RNA-based PE [67]. Thus, optimization of the RNA component discussed in Section 3.3 has direct consequences for the feasibility of transient delivery.

Nonviral nanoparticles provide another emerging route to in vivo PE. Optimized lipid nanoparticle formulations have been used for delivery of genome editor RNPs, including prime editing systems, providing a strategy that combines transient editor exposure with the potential for formulation-dependent tissue targeting [68]. Other protein-based nanoparticle systems are also being developed for cytosolic delivery of nucleic acids and proteins in vivo [69]. These approaches expand the delivery landscape beyond viral vectors, although their relative advantages remain dependent on target tissue, cargo format, route of administration, manufacturing requirements, and the desired duration of editor exposure.

Delivery requirements differ fundamentally between ex vivo and in vivo applications. Ex vivo editing permits direct introduction of editor components into isolated cells and, where appropriate, characterization of the edited population before transplantation. RNP- and mRNA-based PE has been used to generate precisely edited human pluripotent stem cell models [70]. In this setting, intracellular delivery efficiency, cell viability, preservation of cell function, and scalability may be more important than tissue tropism. By contrast, in vivo editing requires the complete editing machinery to reach the relevant tissue and cell population after administration, making biodistribution and cell-specific uptake additional determinants of therapeutic performance.

The multicomponent nature of PE makes this distinction particularly important. PE3-like systems require coordinated delivery of the editor, pegRNA, and secondary nicking guide, whereas twinPE and related paired pegRNA architectures require multiple guide components to be present in the same cell. Repair-modulated systems can introduce still another component. Increasing molecular sophistication can therefore improve editing under optimized experimental conditions while simultaneously increasing the stoichiometric burden of delivery. Compact editors, split proteins, stabilized pegRNAs, RNPs, and alternative carriers address different aspects of this problem rather than constituting a single linear progression toward an optimal delivery platform.

Tissue-specific studies further demonstrate that successful delivery must ultimately be evaluated in biological rather than purely molecular terms. Dual-AAV systems have enabled PE in mouse brain, liver, and heart [17], while other in vivo approaches have extended editing to disease-relevant tissues. Importantly, successful delivery and measurable sequence correction do not themselves establish therapeutic efficacy. A delivery system must reach a sufficient fraction of the disease-relevant cell population and produce enough correctly edited alleles to alter the biological phenotype. This distinction becomes particularly important in the therapeutic studies considered in Section 3.6.

**Figure 6:**
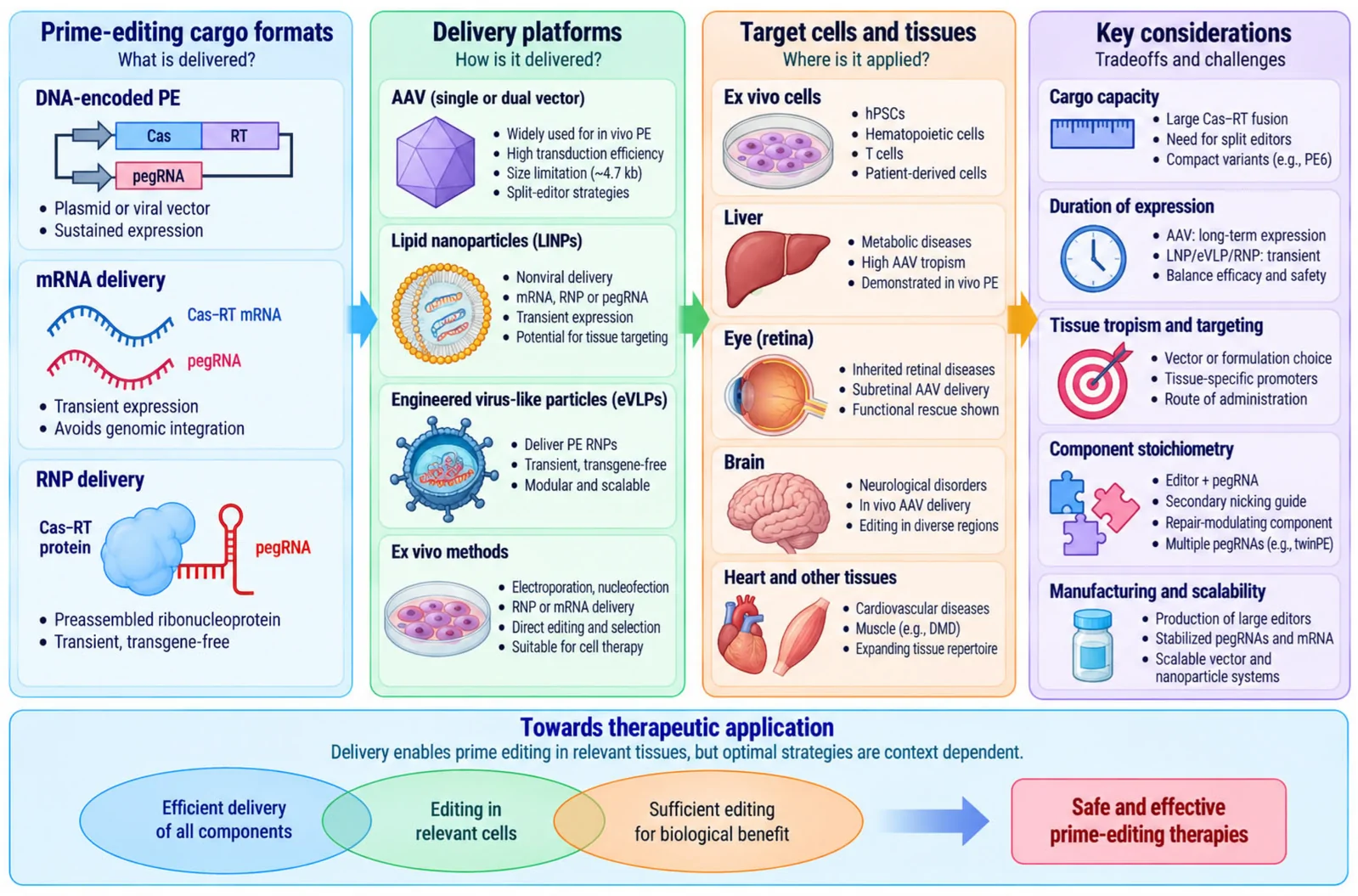
Delivery strategies and translational considerations for prime editing. Prime editing components can be delivered as DNA-encoded systems, mRNA together with pegRNA, or preassembled ribonucleoprotein (RNP) complexes. Major delivery approaches include single- or dual-vector adeno-associated virus (AAV) systems, lipid nanoparticles (LNPs), engineered virus-like particles (eVLPs), and ex vivo methods such as electroporation or nucleofection [17,61,62,63,65,66,68]. Compact prime editors and chemically stabilized pegRNAs provide complementary strategies for overcoming cargo-size and RNA stability limitations [4,64,67]. Delivery requirements vary substantially among ex vivo cells and in vivo tissues, including liver, retina, brain, heart, and muscle. Selection of an appropriate platform therefore requires balancing cargo capacity, duration of editor exposure, tissue tropism, component stoichiometry, manufacturing scalability, and potential for redosing. Efficient delivery and molecular editing are necessary but are not sufficient for therapeutic efficacy; clinically meaningful translation ultimately requires adequate editing of disease-relevant cells to produce biological or functional benefit. Abbreviations: AAV: adeno-associated virus; Cas: CRISPR-associated protein; eVLP: engineered virus-like particle; hPSC: human pluripotent stem cell; LNP: lipid nanoparticle; PE: prime editing; pegRNA: prime editing guide RNA; RNP: ribonucleoprotein; RT: reverse transcriptase.

Collectively, the evidence demonstrates diversification from DNA-encoded and ex vivo PE toward split viral vectors, compact editors, mRNA and RNP delivery, eVLPs, lipid nanoparticles, and tissue-directed in vivo systems. These strategies involve different tradeoffs among cargo capacity, duration of editor exposure, tissue tropism, component stoichiometry, manufacturing complexity, and potential for redosing. The current evidence does not identify a universally optimal delivery platform; rather, delivery must be matched to the editor architecture, target tissue, and therapeutic objective. Accordingly, delivery should be considered an integral component of prime editor design rather than a downstream technical step. The principal PE cargo formats, delivery platforms, and translational tradeoffs are summarized in Figure 6.

### 3.6. Therapeutic Applications and Functional Rescue

Therapeutic application was the second largest thematic category in the final evidence set, with 90 of 294 studies (30.6%) addressing disease-associated variants, disease models, or potential therapeutic applications of prime editing. The evidence encompassed metabolic, pulmonary, hematologic, neuromuscular, cardiovascular, retinal, neurological, and other inherited disorders. However, these studies represent substantially different levels of translational evidence. A central distinction is therefore required between correction of a disease-associated DNA sequence, restoration of a molecular or cellular function, and rescue of a disease phenotype in vivo. Molecular editing efficiency alone should not be interpreted as evidence of therapeutic efficacy.

Early therapeutic studies established that PE could correct pathogenic variants in patient-derived cells and organoids. Schene et al. demonstrated prime editing-mediated functional repair in patient-derived disease models, providing an early example in which sequence correction was evaluated in a disease-relevant cellular context [71]. Subsequent studies extended PE to induced pluripotent stem cells (iPSCs), organoids, primary cells, and differentiated disease models. For example, PE has been used to generate and correct disease-associated mutations in human iPSCs [72,73] and to investigate mutations associated with ACTB-related developmental disorders [74]. Such studies are important for disease modeling and therapeutic development, but correction of a variant in a cellular model does not by itself demonstrate therapeutic rescue.

Metabolic and liver disorders provided some of the earliest demonstrations of therapeutic PE in vivo. Prime editing was applied to a mouse model of metabolic liver disease, establishing that pathogenic alleles could be targeted directly in an intact mammalian organ [75]. Subsequent work corrected the common PAH pArg408Trp variant associated with phenylketonuria in vivo [76]. These studies illustrate an important principle for interpreting therapeutic editing: relatively modest molecular correction can potentially produce substantial biological benefit when partial restoration of an enzyme is sufficient to alter systemic metabolism. Thus, the therapeutically relevant threshold of editing is disease-dependent rather than a fixed percentage applicable across disorders.

Cystic fibrosis (CF) provides a particularly informative progression from genomic correction toward functional rescue. Prime editing has been used to correct CFTR mutations in human organoids and airway epithelial cells, with restoration of CFTR activity providing a functional endpoint beyond sequence correction [77]. Systematic optimization of editor architecture, pegRNA design, and DNA repair modulation subsequently improved correction of the common CFTR F508del mutation in primary human airway epithelial cells [78]. Together, these studies demonstrate why functional measurements are particularly important in therapeutic PE: the clinically relevant question is not simply whether the CFTR sequence was corrected, but whether sufficient chloride channel function was restored in disease-relevant airway cells.

Hematologic disorders represent another important therapeutic setting because hematopoietic cells can potentially be edited ex vivo, characterized, and returned to the patient. PE has been applied to pathogenic globin variants and regulatory sequences relevant to hemoglobinopathies. Recent work used PE to convert repressor motifs in the HBG1/2 promoters into activator motifs, increasing γ-globin expression while addressing sickle cell-associated biology [79]. This strategy illustrates that therapeutic PE need not necessarily recreate the wild-type sequence. In some diseases, installation of a protective or compensatory genotype may provide an alternative route to functional benefit.

Neuromuscular disorders further demonstrate the distinction between sequence correction and restoration of disease-relevant protein function. Duchenne muscular dystrophy (DMD) is particularly challenging because pathogenic variants are diverse and therapeutically meaningful correction ultimately requires restoration of dystrophin in sufficient muscle tissue. Early work demonstrated precise correction of DMD-associated exon deletion mutations using prime and base editing [80]. Subsequent high-capacity adenovector delivery of advanced PE systems achieved selection-free gene repair accompanied by rescue of dystrophin synthesis in DMD muscle cells [81]. Additional studies have extended PE to point mutations associated with muscular disorders [82]. Restoration of a missing disease-relevant protein represents stronger therapeutic evidence than DNA correction alone, although rescue in cultured cells remains distinct from functional recovery of muscle in vivo.

Cardiovascular applications similarly extend PE into disease-relevant differentiated cells. Correction of the cardiomyopathy-associated RBM20-P633L mutation has been demonstrated in human cardiomyocytes [83]. Such studies are translationally important because cardiomyocytes are post-mitotic and are not readily amenable to conventional ex vivo replacement strategies. Consequently, eventual therapeutic application requires efficient delivery to the heart, directly linking disease correction to the delivery constraints discussed in Section 3.5.

Some of the strongest evidence for organ-level functional rescue comes from retinal disease. Qin et al. combined PAM-relaxed PE with in vivo delivery to correct a pathogenic mutation in degenerating mouse retina [84]. Importantly, the study extended beyond measurement of genomic editing to restoration of disease-relevant protein function, preservation of photoreceptors, improved retinal physiological responses, and improved vision-dependent behavior. This represents a qualitatively stronger level of therapeutic evidence because genomic correction was linked sequentially to molecular, tissue, physiological, and organism-level outcomes.

An even more recent example demonstrates the potential for PE-mediated rescue of a severe neurological disorder. Sousa et al. used in vivo PE to correct pathogenic Atp1a3 variants in mouse models of alternating hemiplegia of childhood [85]. AAV9-mediated editing in the central nervous system restored ATPase activity and improved clinically relevant phenotypes, including paroxysmal episodes, motor abnormalities, and cognitive deficits, while substantially extending lifespan. Independent current source verification confirms that this study linked DNA and RNA correction to ATPase restoration, behavioral improvement, and survival. This study therefore represents one of the clearest examples in the current evidence set of a complete progression from molecular correction to organism-level functional rescue.

The therapeutic category also includes studies that should not be interpreted as therapeutic rescue. PE has been used to construct isogenic models of Mendelian diseases [73], generate disease-associated variants, develop mutation-specific reporters [86], and perform high-throughput functional interrogation of human genetic variants [87]. These studies contribute directly to therapeutic development and functional genomics but primarily demonstrate technological or disease modeling proof of concept, rather than treatment of an existing disease phenotype. Maintaining this distinction is important when assessing the translational maturity of the PE literature.

**Figure 7:**
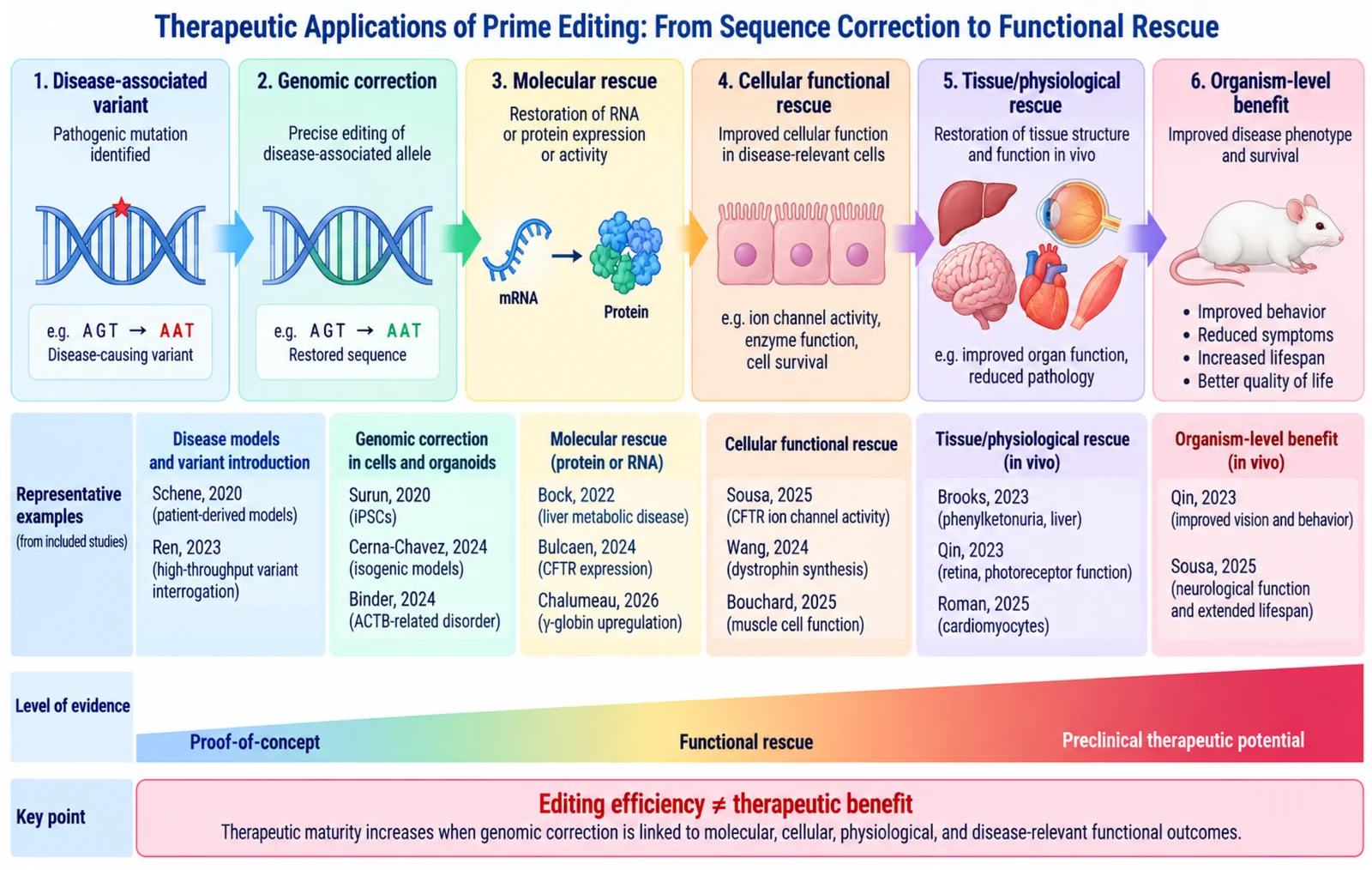
Hierarchy of evidence for therapeutic applications and functional rescue with prime editing. Therapeutic evidence for prime editing (PE) can be considered as a progression from identification of a disease-associated variant through genomic correction, molecular rescue, cellular functional rescue, tissue or physiological rescue, and organism-level phenotypic benefit. Representative studies from the systematic evidence set illustrate different positions along this continuum, including correction in patient-derived and isogenic disease models [71,72,73]; restoration of disease-relevant molecular or cellular functions in metabolic, pulmonary, hematologic, and neuromuscular disorders [75,77,79,81]; and in vivo physiological or organism-level rescue in phenylketonuria, retinal degeneration, and neurological disease [76,84,85]. The increasing evidence gradient emphasizes that editing efficiency is not equivalent to therapeutic benefit: demonstration of sequence correction alone represents therapeutic proof of concept, whereas stronger preclinical evidence links genomic correction to molecular restoration, disease-relevant cellular or tissue function, and ultimately improvement in physiological or organism-level phenotypes. The examples shown are illustrative rather than exhaustive and do not imply that every study within a given disease category has achieved the same level of therapeutic evidence. Abbreviations: iPSC: induced pluripotent stem cell; mRNA: messenger RNA; PE: prime editing.

Accordingly, the therapeutic evidence can be organized as a progression of increasingly stringent endpoints: (i) editing of a disease-associated sequence; (ii) restoration of RNA or protein expression; (iii) rescue of disease-relevant cellular function; (iv) tissue or physiological rescue in vivo; and (v) improvement of organism-level disease phenotypes or survival. Studies such as CFTR correction in airway models [77,80] and restoration of dystrophin synthesis [81] demonstrate functional rescue beyond DNA correction, whereas the retinal study of Qin et al. [84] and neurological study of Sousa et al. [85] extend this evidence to organism-level phenotypic improvement. This hierarchy is summarized in Figure 7.

This framework also clarifies why editing efficiency and therapeutic benefit are not equivalent. The biological consequences of a given editing percentage depend on the disease mechanism, target tissue, distribution of edited cells, dominance or recessivity of the pathogenic allele, protein turnover, and the fraction of corrected cells required to alter physiology. Conversely, relatively modest editing may be therapeutically meaningful when partial restoration of an enzyme or secreted product is sufficient to correct disease physiology. The current PE literature therefore does not support a universal editing efficiency threshold for therapeutic success.

Collectively, the 90 studies classified as therapeutic demonstrate substantial progression from correction of pathogenic variants in cultured cells to functional studies in patient-derived cells and organoids and, in selected cases, meaningful phenotypic rescue in animal models. Nevertheless, the evidence remains predominantly preclinical, and therapeutic maturity varies markedly among applications. The strongest evidence connects the complete translational chain—delivery → genomic correction → molecular restoration → cellular or physiological recovery → disease-relevant functional benefit. Figure 7 uses this hierarchy rather than editing percentage alone to distinguish therapeutic proof of concept from increasingly compelling evidence of functional rescue.

### 3.7. Computational and AI-Guided Design

Computational approaches have become increasingly important as the prime editing design space has expanded. In the final evidence set, 15 of 294 studies (5.1%) were classified as computational or AI-related. Although this represents a relatively small fraction of the literature, computational methods have progressed from pegRNA efficiency prediction to models incorporating edit type, DNA repair determinants, chromatin context, and editor architecture, and more recently to AI-guided engineering of PE proteins. Thus, computational approaches increasingly influence not only which editing strategy is selected but also how components of the editing system are designed. The corrected reference placement map identifies a concentrated group of studies addressing computational and AI-guided PE design.

The first major computational challenge was selection of effective pegRNAs. A single intended edit can often be generated using numerous combinations of spacer sequence, primer binding site (PBS), reverse transcription template (RTT), and secondary nicking guide, yet editing efficiencies can vary markedly among candidate designs. Early systematic experimental analysis identified sequence determinants associated with pegRNA activity and enabled prediction of editing efficiencies in human cells [88]. Easy-Prime subsequently incorporated machine learning into an integrated PE design framework [89]. These studies established the principle that empirical PE datasets could be converted into predictive tools capable of reducing the number of pegRNAs requiring experimental screening.

Increasingly large experimental datasets subsequently enabled prediction across a broader range of edits and outcomes. Mathis et al. developed deep learning models to predict both PE efficiency and product purity [90], while sequence features and DNA repair determinants were used to predict prime editing insertion efficiencies [91]. Prediction was also extended across diverse PE architectures and cell types, emphasizing that guide performance depends on the combination of pegRNA, editor configuration, and cellular environment rather than pegRNA sequence alone [92]. Transformer-based approaches such as DTMP-prime represent a further expansion of machine learning methods for predicting pegRNA activity [93].

An important subsequent development was incorporation of genomic and chromatin context. Machine learning models trained across diverse chromatin environments demonstrated that local chromatin state contributes information beyond pegRNA sequence alone [94]. PRIDICT2.0 and ePRIDICT further formalized systematic pegRNA design across different experimental contexts [95]. Graph-based neural network approaches have similarly integrated sequence and structural features for prediction of genome editing efficiency [96]. Collectively, these approaches support a transition from sequence-only prediction toward models in which editing performance emerges from interactions among pegRNA architecture, genomic sequence, chromatin environment, and cellular processing of the editing intermediate.

Computational tools also facilitate experimental scale. Automated platforms such as PEGG support high-throughput generation of prime editing sensor libraries [97], whereas EditABLE integrates computational tools to assist selection and design of genome editing strategies for specified applications [98]. These approaches are particularly relevant to saturation genome editing and large-scale variant interrogation, where empirical optimization of every candidate pegRNA would be impractical. Computational design therefore serves two related purposes: improving individual PE experiments and enabling PE to function as a scalable platform for interrogation of large numbers of genetic variants.

Specificity represents a complementary computational problem. TAPE-seq was developed as a cell-based approach for predicting genome-wide off-target effects of prime editors [99], while computational analysis of RNA–DNA interaction fingerprints has provided a broader framework for genome-wide CRISPR off-target prediction [100]. These studies emphasize that prediction of high intended editing and prediction of acceptable specificity are distinct objectives. A pegRNA predicted to produce high on-target efficiency is not necessarily optimal if it also increases unwanted products or off-target activity. Future design systems will therefore benefit from multi-objective optimization incorporating efficiency, product purity, specificity, and genomic integrity rather than ranking candidates solely by intended edit frequency.

Cas engineering also contributes directly to the computational design space of PE because PAM availability constrains both target accessibility and the geometry between the nick and intended edit. Within the PE-specific evidence set, PAM-flexible SpCas9 variants expanded the range of sequences accessible to prime editing [28], while Cas12a-based PE architectures provided distinct PAM requirements and editing geometries [29]. These findings demonstrate that selection of an optimal PE strategy should consider Cas architecture together with pegRNA design, genomic context, and the position of the intended edit.

Computational design is also beginning to address cellular determinants of editing. An AI-generated MLH1-binding protein was developed to inhibit mismatch repair and improve PE, demonstrating that computational protein design can be directed toward DNA repair components as well as Cas proteins and pegRNAs [25]. This approach links AI-guided design directly to the repair modulation strategies described in Section 3.4.

The most direct transition from computational prediction to editor engineering is represented by AI-guided RT redesign. Tao et al. used structure-informed AI to redesign laboratory-evolved RTs after identifying a tradeoff in which some activity-enhancing mutations compromised protein stability and expression [5]. This strategy produced the PE8 family, in which computationally guided redesign was used to improve the balance among RT activity, protein stability, expression, and editing performance at the evaluated targets. PE8 therefore represents a qualitatively different use of AI from pegRNA ranking: rather than predicting which existing configuration is likely to work, computational design contributes directly to construction of the editing protein.

Taken together, the computational literature demonstrates a progression: empirical screening → predictive modeling → context-aware prediction → AI-guided molecular design. Nevertheless, predictive accuracy should not be equated with experimental or therapeutic validation. Models remain dependent on the datasets on which they were trained and may not generalize equally to new cell types, genomic contexts, editor architectures, delivery formats, or edit classes. Computationally prioritized designs therefore remain experimentally testable predictions. The emerging paradigm is instead an iterative design–build–test–learn cycle in which high-throughput experimental data train predictive models, computational approaches nominate improved guides or proteins, and experimental validation generates new information for subsequent model refinement. This integration of computation with experimentation may ultimately permit simultaneous optimization of pegRNA, Cas protein, RT, repair environment, specificity, and delivery-compatible architecture.

### 3.8. Emerging and Large-Scale Genome Editing

A major frontier in PE is the transition from local sequence correction toward programmable genome writing at increasingly large scales. Conventional single-pegRNA PE is particularly well suited to substitutions and relatively small insertions and deletions, whereas many disease-associated alterations involve larger deletions, duplications, repeat-associated changes, structural variants, or loss of an entire functional sequence. The systematic evidence set therefore contained a technologically diverse group of studies investigating paired pegRNAs, large deletions and replacements, recombinase-assisted integration, expanded editing geometries, alternative polymerases, and other emerging genome writing approaches. The S4 mapping identifies 17 studies principally associated with this category. Importantly, these approaches differ substantially in mechanism and experimental maturity and should not be interpreted as equivalent stages of a single technological progression.

One of the earliest routes beyond conventional single-pegRNA editing was coordination of paired PE reactions. Guide RNA pairs were used to increase the efficiency and precision of selected edits [101], while PRIME-Del demonstrated programmable genomic deletions using paired prime editing reactions [102]. Related paired approaches have subsequently extended PE to additional structural modifications. These studies established the broader principle that editing intermediates generated at two genomic positions can be coordinated to produce alterations beyond the practical scope of a conventional single pegRNA.

This principle was substantially extended by twin prime editing (twinPE). TwinPE uses two pegRNAs targeting opposite DNA strands to generate complementary edited intermediates, enabling programmable deletion, replacement, integration, and inversion of substantially larger DNA sequences [6]. Rather than simply increasing the efficiency of conventional PE, twinPE changes the architecture of the editing reaction and provides a programmable intermediate that can also be coupled to other genome engineering enzymes. It therefore represents an important bridge between local PE and large-scale genome writing.

PE has subsequently been combined with site-specific integrases and recombinases to expand cargo capacity. A prominent example is PASTE, which couples prime editing-mediated installation of an integration site with a serine integrase to enable targeted insertion of large DNA cargo [103]. The original PASTE study was identified during peer review rather than through the systematic search because the exact search term “Prime Editing” was not present in its searchable title/abstract terminology; it is therefore cited here as contextual evidence and is not included in the 294-study systematic evidence set [103]. In these hybrid architectures, PE establishes a programmable genomic sequence or landing site that is subsequently recognized by another enzyme capable of integrating substantially larger DNA cargo.

PrimeRoot provides an additional example of this broader technological concept in plants, combining dual prime editing with Cre-mediated site-specific recombination to enable programmable sequence integration [104]. Because the present review prespecified crop-, agricultural-, and plant-focused studies as outside its eligibility criteria, the original PrimeRoot study was not included in the 294-study systematic evidence set. It is cited here as contextual evidence because of its relevance to the technological development of recombinase-assisted prime editing architectures [104].

Continuous evolution of recombinases coupled to prime editing-assisted integration generated a strategy for efficient site-specific integration of large genes in mammalian cells [34]. Protocols have subsequently formalized the integration of large genetic payloads using PE together with site-specific integrases [105]. TwinPE-derived strategies have likewise been applied to insertion of recombinase recognition sequences [106] and, more recently, to whole-gene humanization through twinPE and evolved Bxb1-mediated cassette exchange.

The distinction between these hybrid approaches and conventional PE is important. In conventional PE and twinPE, reverse transcription of pegRNA-encoded information contributes directly to formation of the intended edited sequence. In recombinase- or integrase-assisted architectures, PE can instead create the genomic substrate required for a subsequent large-cargo integration reaction. Such technologies are therefore appropriately considered PE-enabled genome writing systems, but the entire large DNA payload is not necessarily synthesized by the conventional pegRNA–RT reaction. Maintaining this distinction prevents large-cargo systems from being presented as simply higher-capacity versions of PE1–PE8.

Several recent approaches have increased the number or geometry of programmable PE components. EXPERT was developed to expand PE efficiency and the range of large-fragment edits by overcoming constraints imposed by the conventional position of the pegRNA-directed nick [36]. This work emphasizes that edit size is not the only constraint on PE: PAM availability, strand orientation, nick position, and the geometry of the intended alteration can determine whether a large sequence change is accessible.

An additional expansion is represented by quadruple-pegRNA editing, which coordinates four pegRNAs to support programmable large genomic insertion [37]. This strategy extends the paired-guide concept by increasing the number of programmable RNA components participating in construction of the edited locus. Its development illustrates how PE is evolving from a single-guide editing reaction toward multicomponent genome writing systems, although the increasing number of required components simultaneously increases the delivery and stoichiometric challenges discussed in Section 3.5.

Other recent strategies use PE-generated intermediates or complementary DNA repair mechanisms to accomplish large DNA replacement or integration. Prime editing-mediated microhomology has been used to facilitate efficient replacement of large genomic DNA segments [35]. Nonviral targeted integration of large DNA has also been demonstrated in primary human T cells without requiring conventional DSB-mediated integration [107]. These approaches are particularly relevant to therapeutic cell engineering because they begin to combine the programmability of precision editing with cargo sizes traditionally associated with gene addition strategies.

Large-scale PE-related approaches are not restricted to insertion and replacement. They increasingly encompass structural genome engineering, including inversions. Prime editing-based inversion strategies have been developed to generate precise genomic inversions over increasingly large sequence intervals [38], while twinPE-assisted cassette exchange strategies provide another route toward replacement or humanization of larger endogenous genomic regions. These capabilities are potentially important because many clinically relevant variants cannot be reduced to single-nucleotide substitutions or small indels.

A mechanistically distinct emerging direction replaces the reverse transcriptase-centered reaction with DNA-dependent DNA polymerase (DNAP) activity. Liu et al. demonstrated targeted genome editing using a DNA-dependent DNA polymerase and exogenous DNA-containing templates [40]. Chimeric oligonucleotide-directed editing (CODE) subsequently provided another architecture coupling programmable targeting to DNA polymerase-mediated synthesis [39]. These approaches are conceptually related to PE because they combine programmable Cas-mediated targeting with templated DNA synthesis, but they should not be presented as established successors to RT-based PE. Indeed, only two of the 294 included studies (0.7%) were classified as polymerase-related, underscoring the early stage of this branch of the evidence base.

Cellular DNA repair remains relevant even as the scale of programmable editing increases. Dual inhibition of DNA-PK and polymerase θ has been reported to improve the precision of multiple PE configurations, including paired and twinPE-related systems [60]. Thus, increasing the synthetic capacity of the editor does not eliminate dependence on cellular processing of PE intermediates. On the contrary, larger and more complex DNA intermediates may increase the importance of understanding how repair pathways determine productive editing versus unwanted products.

Several additional technologies in the mapped evidence set illustrate the broader transition toward large-scale genome engineering, including methods for large-DNA knock-in and cassette exchange [108,109,110]. These studies are useful for placing PE-enabled genome writing in the broader technological landscape, but they should be distinguished from systems in which PE itself is the central editing mechanism. This distinction is particularly important given the review’s focus on PE rather than large-fragment genome engineering generally.

Expansion of edit scale also creates additional requirements for product validation. For a single-nucleotide edit, local sequencing may provide substantial information about editing outcome. For a kilobase-scale insertion, replacement, or inversion, demonstration of a correct local junction is insufficient to establish integrity of the complete product. Evaluation increasingly requires confirmation of both junctions, full-length sequence integrity, orientation, copy number, unintended integrations, structural rearrangements, and broader genomic integrity. Thus, the metrics used to evaluate conventional PE cannot simply be transferred unchanged to large-scale genome writing.

The same principle applies to therapeutic interpretation. A larger programmable edit does not necessarily represent a more advanced therapeutic technology. Increasing edit size commonly requires additional pegRNAs, donor molecules, integrases or recombinases, and more complex intracellular coordination, thereby increasing the delivery and stoichiometric burden. The emerging systems also vary markedly in the number of laboratories, targets, cell types, and in vivo settings in which they have been independently evaluated. Consequently, editing scope and technological or therapeutic maturity should be considered separate dimensions.

Taken together, the evidence demonstrates a transition from local sequence rewriting toward increasingly versatile forms of programmable genome writing. Paired PE and PRIME-Del established coordinated two-site editing [101,102]; twinPE expanded programmable deletion, replacement, integration, and inversion [6]; recombinase-assisted strategies increased the potential scale of targeted gene integration [34]; EXPERT expanded editing geometry [36]; quadruple-pegRNA editing increased programmable insertion capacity [37]; and recent approaches extended large-fragment replacement and integration [38,107] and large genomic inversions [38]. Alternative DNAP-based architectures further demonstrate that templated precision editing may eventually extend beyond conventional RT-based PE [40,41].

**Figure 8:**
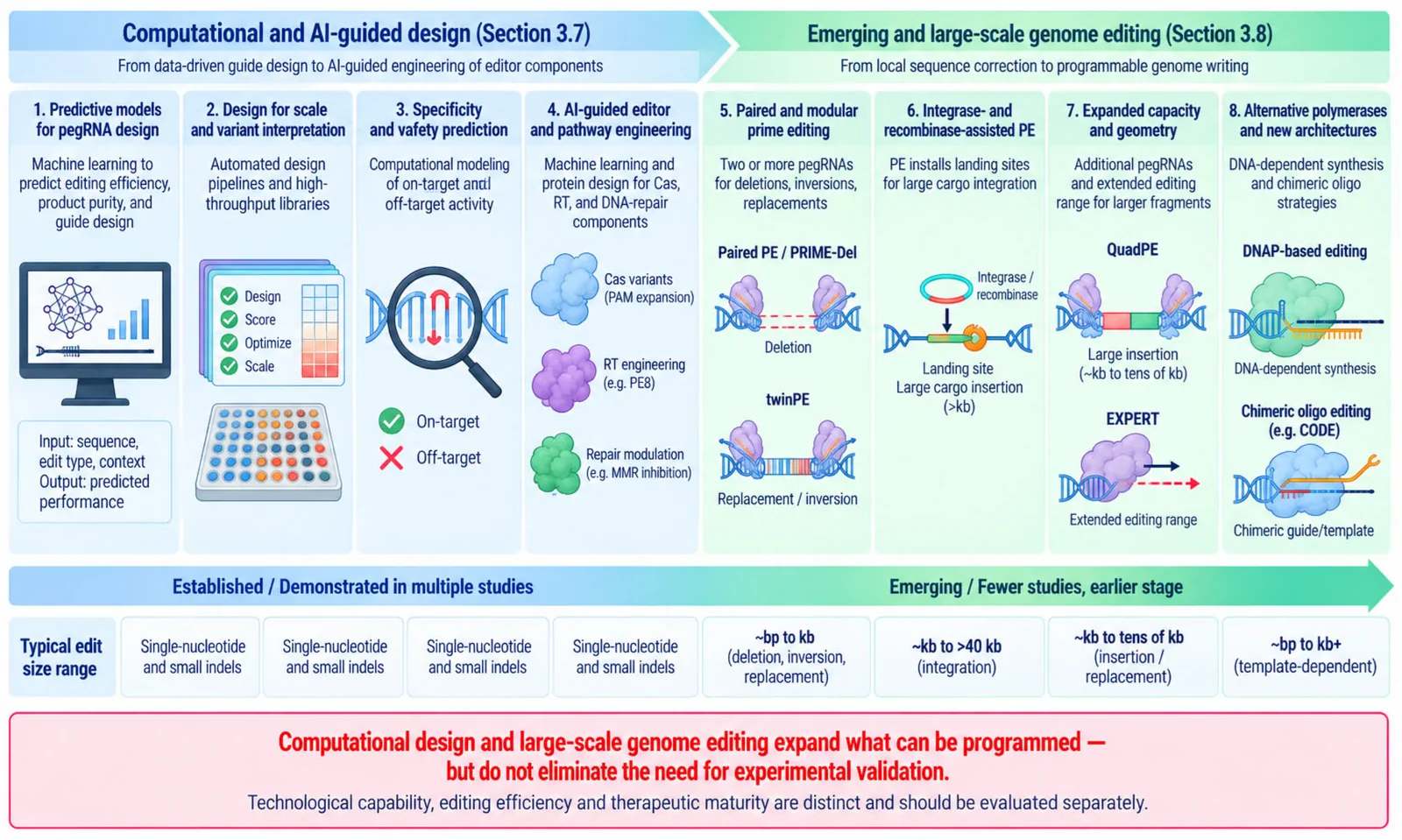
Computational design and expansion of the prime editing design space. Computational and AI-guided approaches support pegRNA prediction, scalable experimental design, specificity assessment, and engineering of PE components [5,88,90,94]. Emerging architectures extend PE toward larger and more complex genomic alterations through paired PE, twinPE, integrase/recombinase-assisted systems, expanded editing geometries, and alternative polymerases [6,34,36,37,40,102]. These technologies expand the PE design space but vary substantially in experimental validation and therapeutic maturity. Abbreviations: AI: artificial intelligence; DNAP: DNA-dependent DNA polymerase; PE: prime editing; pegRNA: prime editing guide RNA; RT: reverse transcriptase; twinPE: twin prime editing.

These technologies collectively broaden the conceptual boundaries of PE, but they differ substantially in mechanism, experimental validation, delivery requirements, and translational maturity. Accordingly, newer architectures should be evaluated by what has been experimentally demonstrated rather than by their theoretical editing capacity. Together with the computational developments described in Section 3.7, these emerging technologies illustrate two complementary expansions of the PE design space: computational and AI-guided methods are increasingly optimizing how editors are designed, whereas large-scale genome writing systems are expanding what types and sizes of genomic alterations can be programmed. These complementary developments are summarized in Figure 8.

## 4. Discussion

This systematic review maps the development of prime editing from its introduction in 2019 through 11 August 2026. Across the 294 studies included in the final evidence synthesis, PE has evolved from a defined Cas9 nickase–reverse transcriptase (RT) system into a modular genome editing platform in which the Cas protein, RT, pegRNA, DNA repair environment, and delivery strategy can each be modified. The evidence therefore supports a multidimensional rather than linear model of PE development. Later-numbered editors should not be assumed to be universally superior; performance depends on the intended edit, genomic target, pegRNA design, cell type, delivery method, and desired balance between efficiency and product purity.

Three conclusions emerge from the evidence. First, optimization of PE components is increasingly interdependent. pegRNA structure and stability influence the substrate presented to the RT; Cas selection determines target accessibility and nick geometry; cellular repair pathways influence resolution of editing intermediates; and delivery determines whether an optimized editor reaches disease-relevant cells. Second, editing efficiency is not equivalent to therapeutic benefit. Sequence correction provides evidence of molecular editing, whereas stronger therapeutic evidence requires restoration of molecular or cellular function and, ultimately, improvement in tissue physiology or disease phenotype. Third, computational design and emerging large-scale editing architectures are expanding both how PE systems are designed and the types of genomic alterations that can be programmed. These advances increase the potential scope of PE while simultaneously increasing requirements for delivery, genomic safety, and functional validation.

### 4.1. Prime Editing as a Modular Genome Editing Platform

The original PE system established the core nCas9–RT–pegRNA architecture and PE1–PE3b configurations [1]. Subsequent developments demonstrate that essentially every component of this architecture can be optimized. Mismatch repair modulation contributed to PE4, PE5, and PEmax [2]; stabilization of the pegRNA 3′ extension generated epegRNAs [3]; protein evolution produced PE6 variants [4]; recruitment of the endogenous RNA-binding protein La led to PE7 [18]; and AI-guided RT redesign generated PE8 [5]. These developments are compared in Table 1 and support viewing PE as a collection of interacting functional modules rather than a single progressively improved editor.

This modular interpretation explains why direct ranking of PE architectures is difficult. Secondary strand nicking can increase intended editing but may alter indel formation and product purity, while repair modulation can substantially increase editing for some targets but has context-dependent effects [1,2]. Similarly, evolved RTs, pegRNA stabilization, PAM-flexible Cas proteins, and alternative Cas architectures address different constraints rather than a common limiting step. Consequently, the optimal configuration depends on the biological and technical requirements of the individual application.

Architectural diversification also extends the scale of programmable editing. TwinPE uses paired pegRNAs to enable larger deletions, replacements, integrations, and inversions [6], while newer paired and hybrid approaches further expand the range of possible genomic alterations. These systems move PE toward programmable genome writing, but they also illustrate an important distinction: greater editing capability does not necessarily indicate greater efficiency, safety, or therapeutic maturity.

### 4.2. Efficiency, Product Purity, and Genomic Safety

Avoidance of an intentionally introduced double-strand break is an important advantage of PE, but it does not eliminate unintended editing outcomes. The evidence indicates that intended edit efficiency, product purity, specificity, and genomic integrity should therefore be considered together rather than as independent endpoints.

Cellular DNA repair provides a particularly clear example. Mismatch repair can oppose productive PE, and transient MMR inhibition can substantially increase editing, forming the basis of PE4 and PE5 [2]. Additional repair pathways, including DNA-PK- and Polθ-dependent processes, can also influence PE outcomes [60]. However, because these pathways contribute to normal genome maintenance, therapeutic manipulation requires consideration of the duration and cellular specificity of repair inhibition as well as its effect on editing efficiency.

Product quality is similarly important. PE can generate unintended on-target products, indels, incomplete sequence incorporation, or pegRNA-associated products, while PAM-relaxed and increasingly active editors require careful assessment of off-target activity [7,8,9,10]. These concerns become increasingly important for larger sequence alterations, for which confirmation of a correct local junction alone may not establish the integrity of the complete edited locus.

The field would therefore benefit from more standardized reporting of PE outcomes. A therapeutically relevant assessment should include intended editing, unwanted on-target products, off-target activity, genomic integrity, and cellular function. A system producing somewhat lower intended editing but greater product purity and limited editor exposure may ultimately be preferable to one producing a higher editing percentage accompanied by undesirable genomic outcomes. Thus, maximum editing efficiency alone is an insufficient criterion for identifying an optimal therapeutic editor.

### 4.3. Delivery and Functional Rescue as Translational Bottlenecks

Delivery remains a major barrier separating molecular optimization from therapeutic application. The large Cas–RT fusion, pegRNA, and, for some architectures, additional guide RNAs or regulatory components create substantial cargo and stoichiometric challenges. Viral, nonviral, RNP, mRNA, and ex vivo approaches each address different aspects of this problem, but the evidence does not identify a universally optimal delivery platform.

AAV-based approaches have demonstrated in vivo PE while highlighting the cargo size constraint of the technology [17,62,63]. Compact editor architectures can partially address this limitation [4,64]. In parallel, eVLPs, chemically stabilized pegRNAs, and nonviral delivery approaches offer opportunities for transient editor exposure [65,66,67,68]. These findings indicate that editor and delivery engineering should increasingly be performed together.

The optimal delivery strategy will also be disease- and tissue-dependent. Ex vivo editing permits characterization of edited cells before administration and may be particularly suitable for transplantable cell populations. Local delivery may facilitate applications in tissues such as the retina, whereas widespread neurological, cardiac, or skeletal muscle diseases impose substantially greater distribution requirements. Thus, the most active molecular editor may not necessarily be the most clinically useful if its architecture is incompatible with delivery to the relevant tissue.

The therapeutic evidence further demonstrates why delivery and editing efficiency are intermediate rather than final endpoints. Among the included therapeutic studies, evidence ranges from correction of pathogenic sequences to restoration of molecular function, cellular rescue, physiological improvement, and organism-level phenotypic benefit. Functional restoration has been demonstrated in disease-relevant models including cystic fibrosis and Duchenne muscular dystrophy [77,78,80,81], while selected in vivo studies have linked genomic correction to physiological or organism-level improvement [84,85].

The fraction of cells requiring correction will vary substantially among diseases according to tissue organization, disease mechanism, protein turnover, dominance or recessivity, and whether corrected cells can provide benefit to neighboring cells. Therapeutic PE should therefore be judged using disease-specific functional endpoints rather than a universal editing efficiency threshold.

### 4.4. Computational Design and Expansion of the Editing Space

The growing number of editable variables creates a combinatorial design problem. A single target may require selection among Cas proteins, PAMs, nick positions, PBS and RTT configurations, secondary nicking strategies, RT architectures, repair modulation approaches, and delivery formats. Computational approaches are consequently becoming increasingly important for navigating this design space.

Early computational approaches focused primarily on pegRNA design and prediction of editing efficiency [88,89]. Subsequent models incorporated larger datasets, product purity, DNA repair determinants, and genomic or chromatin context [90,92,94,95]. More recently, computational methods have expanded from predicting experimental performance to engineering components of the editing system itself. Machine learning has been applied to Cas discovery and PAM engineering, while AI-guided protein design has been used to manipulate MMR and redesign RTs [5,25].

PE8 is particularly illustrative of this transition because AI-guided structural redesign was used to balance RT activity, stability, expression, and editing performance [5]. However, computational prediction and AI-guided design should not be equated with biological or therapeutic validation. Models remain dependent on the diversity and quality of their training data, and performance may not generalize across genomic targets, cell types, editor architectures, or delivery conditions.

A future objective should therefore be multi-objective computational optimization rather than prediction of intended edit percentage alone. Models that integrate efficiency, product purity, specificity, genomic integrity, editor size, cellular context, and delivery constraints could support iterative design–build–test–learn workflows. Such approaches may become increasingly valuable as PE expands toward larger and more complex sequence modifications.

### 4.5. Limitations and Priorities for Future Translation

Several limitations should be considered when interpreting this review. First, the field is evolving rapidly: 127 of the 294 included studies (43.2%) were published in 2025 or during the first eight months of 2026. The review therefore represents a time-defined assessment through 11 August 2026 rather than a static evidence base.

Second, substantial methodological heterogeneity precluded meaningful quantitative pooling. Studies differed in editor architecture, pegRNA design, target sequence, intended edit, cell type, delivery method, sequencing strategy, and reported outcome. Systematic mapping and qualitative evidence synthesis were therefore more appropriate than meta-analysis, and editing percentages reported across different studies should not be interpreted as directly comparable.

Third, translational maturity remains uneven. Much of the evidence derives from cultured cells and experimental models, with fewer studies demonstrating functional rescue in primary human cells, disease-relevant animal models, or direct in vivo therapeutic settings. Methods used to identify off-target effects, unintended on-target products, and structural alterations also varied among studies. Absence of a reported adverse editing outcome therefore does not establish that it was comprehensively excluded.

Fourth, the thematic categories used in this review were intentionally non-mutually exclusive and describe overlapping features of the literature rather than independent study populations. In addition, searches were restricted to PubMed and Web of Science, the review was not prospectively registered, and a single conventional risk-of-bias instrument was not applicable across the heterogeneous technological, computational, cell-based, animal, delivery, and therapeutic studies. All 478 candidate records were nevertheless manually re-screened against the eligibility criteria during the final audit, resulting in the 294-study final evidence set.

Finally, emerging large-scale architectures should be interpreted according to what has been experimentally demonstrated rather than their theoretical editing capacity. TwinPE and newer paired, integrase/recombinase-assisted, large-fragment, and alternative polymerase approaches substantially expand the possible scale of genome modification [6,34,36,37,40]. However, these systems generally have less extensive validation than conventional PE and face additional challenges involving efficiency, delivery, product characterization, and genomic integrity.

The next phase of PE development should therefore emphasize standardized reporting of intended and unintended products, comprehensive genomic integrity assessment, validation across diverse primary cells and in vivo models, delivery systems matched to clinically relevant tissues, and disease-specific functional endpoints. Prospective comparisons of competing architectures under common experimental conditions would be particularly valuable. Together, these priorities shift the central translational question from whether PE can install a desired sequence change to whether an appropriately designed and delivered edit can safely produce meaningful biological benefit.

## 5. Conclusions

This systematic review of 294 studies demonstrates that prime editing has evolved rapidly since its introduction in 2019 from a defined Cas9 nickase–reverse transcriptase editing system into a diverse and increasingly modular platform for precision genome modification. The evidence indicates that this development is best understood not as a linear progression of increasingly superior PE generations, but as the diversification of interacting components—including Cas proteins, pegRNAs, reverse transcriptases, DNA repair modulation, and delivery systems—that can be combined according to the requirements of a particular genomic target and biological application.

The therapeutic literature is promising but remains predominantly preclinical. A central conclusion of this review is that editing efficiency should not be equated with therapeutic efficacy. Sequence correction represents an essential first step, but stronger evidence requires demonstration that editing restores molecular function, produces disease-relevant cellular or physiological rescue, and ultimately improves organism-level phenotypes. The appropriate threshold for therapeutic editing is therefore disease- and tissue-dependent rather than a universal percentage of corrected alleles.

Delivery remains one of the most important barriers to clinical translation. The size and multicomponent nature of PE have stimulated complementary solutions including compact editors, split viral systems, mRNA and chemically stabilized pegRNAs, lipid nanoparticles, eVLPs, and ex vivo editing. The diversity of these approaches suggests that future therapeutic PE will likely require co-optimization of the editor and its delivery platform for the target tissue, rather than development of a single universally applicable delivery system.

At the same time, computational and AI-guided approaches are beginning to transform PE from empirical optimization toward predictive design. Models for pegRNA selection, genomic context prediction, specificity assessment, and AI-guided engineering of editor components—including PE8—provide an emerging foundation for integrated editor design [5,88,90]. However, computational prediction remains dependent on experimental training data and must be validated for editing efficiency, product purity, genomic integrity, and biological function.

Finally, paired and hybrid PE architectures are expanding precision editing from individual nucleotide changes toward larger deletions, replacements, integrations, inversions, and potentially gene- or region-scale genome writing [6,34,36,37]. These capabilities are important because many disease genes contain numerous distinct pathogenic variants that could potentially be addressed by replacement of a larger defined genomic region rather than mutation-specific correction. Nevertheless, greater editing capability does not imply greater therapeutic maturity; larger genomic alterations impose correspondingly greater requirements for delivery, product characterization, genomic integrity assessment, and functional validation.

Collectively, the evidence supports a shift in the central question for prime editing. Rather than simply asking which editor produces the highest editing efficiency, the field is moving toward determining what genomic alteration is required to restore biological function and which combination of editor architecture, guide design, cellular context, and delivery system can achieve that outcome safely. This transition—from sequence editing toward outcome-driven precision genome engineering—may ultimately define the role of prime editing in genetic medicine.

## Figures and Tables

**Table 1:** Comparison of major prime editing architectures and emerging extensions.

System	Architecture/Mechanism	Principal Advance	Editing Capability	Efficiency/Product-Quality Considerations	Delivery Implications	Evidence Maturity	Major Limitations	Key Reference(s)
**PE1**	nCas9(H840A)–M-MLV RT + pegRNA	Foundational PE architecture	All 12 base substitutions; small insertions/deletions	Proof-of-principle activity; relatively low efficiency at many targets	Large fusion protein plus pegRNA	Foundational	Limited activity of original RT	[1]
**PE2**	nCas9 fused to engineered M-MLV RT	Increased RT activity	Substitutions; small insertions/deletions	Higher activity than PE1 at tested targets	Similar cargo requirements to PE1	Established	Strong target- and pegRNA-dependence	[1]
**PE3**	PE2 + nicking sgRNA targeting non-edited strand	Biases repair toward edited strand	Same basic edit spectrum as PE2	Often increases intended editing but can increase indels	Requires additional guide RNA	Established	Efficiency–product purity tradeoff	[1]
**PE3b**	Secondary nick preferentially occurs after intended editing	More selective secondary strand nicking	Same basic edit spectrum	Can improve product purity relative to PE3	Additional guide required	Established	Design opportunities are sequence-dependent	[1]
**PE4**	PE2/PEmax-type editing + transient MMR inhibition	Reduces MMR-mediated rejection of edited intermediates	Primarily substitutions and small indels	Can substantially increase editing for MMR-sensitive edits	Adds repair modulation component	Established experimentally	Repair pathway manipulation requires safety consideration	[2]
**PE5**	PE3-type secondary nicking + MMR inhibition	Combines secondary nicking with MMR suppression	Substitutions and small indels	Can increase editing substantially; target-dependent	Additional guide plus repair modulation	Established experimentally	Greater complexity; product profiles require evaluation	[2]
**PEmax**	Optimized editor expression, nuclear localization and architecture	Increased editor activity	Broad conventional PE edit spectrum	Frequently improves activity but is not universally superior	Retains fundamental PE cargo size constraint	Widely used optimization	Performance remains target and cell type-dependent	[2]
**PE6**	Evolved RT and Cas variants	Protein evolution improves activity and/or compactness	Broad PE spectrum; variants optimized for different contexts	Improved performance at evaluated targets; context-dependent	Compact variants can facilitate delivery	Advanced preclinical technology	No single PE6 configuration is optimal for all edits	[4]
**PE7**	Exploits endogenous La-mediated pegRNA stabilization	Improves productive pegRNA utilization	Conventional PE edit spectrum	Can increase editing through RNA stabilization	Does not eliminate major PE cargo constraints	Advanced experimental technology	Benefit remains target and context dependent	[18]
**PE8**	Structure-informed AI-guided redesign of evolved RTs	AI-guided optimization of RT properties	Broad conventional PE spectrum	Improved performance at evaluated targets; not universally superior	Retains multicomponent PE delivery challenges	Emerging advanced architecture	Requires broader independent validation across targets, cells and tissues	[5]
**twinPE**	Two pegRNAs generate complementary edited intermediates	Extends PE to larger and more complex sequence changes	Deletions, replacements, integrations and inversions	Broadens editing scope with increased architectural complexity	Requires two pegRNAs; hybrid applications may require additional components	Established proof-of-concept with expanding applications	Efficiency and delivery become more challenging as edit complexity increases	[6]
**Integrase/recombinase-assisted PE**	PE coupled to site-specific integration/recombination	Extends programmable insertion to larger DNA cargoes	Large-fragment or gene-scale integration/replacement	Expanded edit scale; efficiency and product integrity are system-dependent	Multiple and/or larger components complicate delivery	Emerging	Integration fidelity, cargo delivery and genomic safety require further validation	[34,35]
**Expanded paired/multiplex PE**	Multiple coordinated pegRNAs or altered editing geometries	Expands programmable edit size and geometry	Larger insertions, replacements, deletions and inversions	Performance varies substantially among architectures and targets	Increased component number complicates delivery and stoichiometry	Emerging	Complexity, efficiency, product integrity and scalability	[36,37,38,39]
**Alternative-polymerase approaches**	DNA-dependent DNA polymerase or related alternative templated-editing architectures	Explores mechanisms beyond conventional RT-dependent PE	Alternative templated sequence writing	Evidence remains limited relative to conventional PE	Architecture dependent; delivery requirements not yet standardized	**Early emerging**	Small evidence base; comparative and therapeutic maturity not established	[40,41]

Major PE systems are compared according to molecular architecture, principal advance, demonstrated editing capability, product quality considerations, delivery implications, evidence maturity, and major limitations. Reported editing efficiencies are not directly compared because performance varies substantially with genomic target, pegRNA design, cell type, delivery method, and experimental conditions. Later-numbered PE systems should not be interpreted as universally superior to earlier architectures. Emerging large-scale and alternative polymerase approaches expand the PE design space but currently have less extensive experimental and therapeutic validation than established PE architectures. Abbreviations: AI: artificial intelligence; MMR: mismatch repair; M-MLV: Moloney murine leukemia virus; nCas9: Cas9 nickase; PE: prime editing; pegRNA: prime editing guide RNA; RT: reverse transcriptase; sgRNA: single-guide RNA; twinPE: twin prime editing.

## Data Availability

All data analyzed in this systematic review were derived from publicly available published studies. Study-level information generated during the review and used for evidence classification and synthesis is provided in the Supplementary Materials. No new primary datasets were generated.

## Supplementary Materials

The following supporting information can be downloaded at: https://www.mdpi.com/article/10.3390/genes17091138/s1, Table S1, literature search strategy, study selection audit trail, and characteristics of the 294 included studies; Table S2, evidence extraction and study annotations for the 294 included studies; Table S3, the 184 records excluded during final eligibility assessment and reasons for exclusion; Table S4, reference placement and evidence map for the included studies; and Table S5, completed PRISMA 2020 checklist.

## Abbreviations

**Abbreviation**	**Definition**
AAV	Adeno-associated virus
AD	Alzheimer disease
AI	Artificial intelligence
Cas	CRISPR-associated protein
CF	Cystic fibrosis
CFTR	Cystic fibrosis transmembrane conductance regulator
CNS	Central nervous system
CRISPR	Clustered regularly interspaced short palindromic repeats
DNA-PK	DNA-dependent protein kinase
DMD	Duchenne muscular dystrophy
DSB	Double-strand break
epegRNA	Engineered prime editing guide RNA
eVLP	Engineered virus-like particle
HBB	Hemoglobin subunit beta
HDR	Homology-directed repair
HOPE	Homologous 3′ extension-mediated prime editing
HSPC	Hematopoietic stem and progenitor cell
indel	Insertion or deletion
iPSC	Induced pluripotent stem cell
LNP	Lipid nanoparticle
ML	Machine learning
MLH1	MutL homolog 1
MMR	Mismatch repair
mRNA	Messenger RNA
nCas9	Cas9 nickase
NHEJ	Non-homologous end joining
PAH	Phenylalanine hydroxylase
PAM	Protospacer-adjacent motif
PE	Prime editing/prime editor
PE1	First-generation prime editor
PE2	Second-generation prime editor
PE3	Prime editor with secondary strand nicking
PE3b	PE3 with edit-dependent secondary nicking
PE4	Prime editor with mismatch repair inhibition
PE5	PE3-type editor with mismatch repair inhibition
PE6	Evolved/engineered prime editor family
PE8	AI-redesigned prime editor family
pegRNA	Prime editing guide RNA
PKU	Phenylketonuria
Polθ	DNA polymerase theta
PRIME-Del	Prime editing-mediated deletion
PBS	Primer binding site
RNP	Ribonucleoprotein
RT	Reverse transcriptase
RTT	Reverse transcription template
sgRNA	Single-guide RNA
SNV	Single-nucleotide variant
tRNA	Transfer RNA
twinPE	Twin prime editing
VLP	Virus-like particle
